# Supplementary material for: Metabolic responses of rice cultivars with different tolerance to combined drought and heat stress under field conditions
Source: Gigascience. 2019 May 13;8(5):giz050. doi: 10.1093/gigascience/giz050 (PMC6511916; doi:10.1093/gigascience/giz050)
Supplement: GIGA-D-18-00498_Revision_1.pdf [file giz050_giga-d-18-00498_revision_1.pdf]

GigaScience

Metabolic responses of rice cultivars with different tolerance to combined drought and heat stress under field conditions

--Manuscript Draft--

Manuscript Number:	GIGA-D-18-00498R1
Full Title:	Metabolic responses of rice cultivars with different tolerance to combined drought and heat stress under field conditions
Article Type:	Research
Funding Information:	Bundesministerium für Wirtschaftliche Zusammenarbeit und Entwicklung (Project Number 11.7860.7-001.00; Contract Numbers 81141844 and 81170348) — Dr. Krishna S.V. Jagadish
Abstract:	Background<br><br>Rice is susceptible to both drought and heat stress, in particular during flowering and grain filling, when both grain yield and quality may be severely compromised. However, under field conditions, these two stresses rarely occur separately. Under well-watered conditions, plants avoid heat stress by transpirational cooling, while this is not possible under drought conditions. Although investigating combined drought and heat stress is clearly more agronomically relevant than analyzing the effects of the single stresses, only a few studies of this stress combination, in particular under field conditions, have been published.<br><br>Results<br><br>Three rice cultivars differing in drought and heat tolerance were grown in the field under control and drought conditions in three consecutive years. Drought was applied either during flowering or during early grain filling, and resulted in simultaneous heat stress, leading to reduced grain yield and quality. Analysis by gas chromatography-mass spectrometry (GC-MS) showed distinct metabolic profiles for the three investigated organs (flag leaves, flowering spikelets, developing seeds). The metabolic stress responses of the plants also strongly differed between cultivars and organs. Correlation analysis identified potential metabolic markers for grain yield and quality under combined drought and heat stress from both stress-regulated metabolites and from metabolites with constitutive differences between the cultivars.<br><br>Conclusions<br><br>GC-MS resolved metabolic responses to combined drought and heat stress in different organs of field-grown rice. The metabolite profiles can be used to identify potential marker metabolites for yield stability and grain quality that are expected to improve breeding efforts towards climate change resilient rice.
Corresponding Author:	Dirk K Hincha<br>Max-Planck-Institut fur Molekulare Pflanzenphysiologie<br>Potsdam, GERMANY
Corresponding Author Secondary Information:	
Corresponding Author's Institution:	Max-Planck-Institut fur Molekulare Pflanzenphysiologie
Corresponding Author's Secondary Institution:	
First Author:	Dirk K Hincha
First Author Secondary Information:	
Order of Authors:	Dirk K Hincha
	Lovely Mae F. Lawas

Powered by Editorial Manager® and ProduXion Manager® from Aries Systems Corporation

	Xia Li
	Alexander Erban
	Joachim Kopka
	Krishna S.V. Jagadish
	Ellen Zuther
**Order of Authors Secondary Information:**	

Response to Reviewers:

Reviewer reports:
Reviewer #1: In this manuscript the authors performed metabolic profiling analysis of three rice cultivars grown in field conditions. Drought was applied during early flowering or grain filling. The authors studied the metabolic differences induced by drought and the different responses of the three cultivars. This is a follow up study from the same authors where other agronomic measurements were taken. In this paper the authors make correlations analysis between metabolite levels and yield reduction under stress. I wholeheartedly agree with the authors on the necessity of conducting this type of analysis in plants grown in field conditions and particularly trying to induce several stresses.
My main concern about the paper is about the way the idea of combined drought/heat stress is presented. How plants respond to these two stresses is a very interesting subject and a lot of research is currently going on about how climate change will impact certain areas with a combination or both stresses while in other areas only high temperature will be the predominant factor. In the paper the authors acknowledge the fact that their experimental design only allows them to control drought stress by means of controlling irrigation. Temperature is not controlled in their experimental setup. However, it should be more clear how why the authors consider that the temperature at which they grew the plants in the different seasons is considered stressful for the cultivars used in the experiment. It would be important to clarify this since the main idea of the paper is to study metabolic differences of cultivars grown under a combination of heat and drought stress.

Response: We apologize if we have not made this point sufficiently clear. We have now added information under Data Description to describe how we showed in our earlier publication that the plants were indeed heat stressed. The modified text now reads as follows:
We performed three field experiments in 2013, 2014 and 2015 at the International Rice Research Institute (IRRI) in the Philippines during the dry season (late April to early May, the hottest time of the year) including the three rice cultivars N22, Dular and Anjali that differ in their response to drought and heat stress. Plants were either grown in flooded paddies throughout their life cycle, or were drought stressed either during flowering or early grain filling. At the end of the stress period, plants were rewatered to allow seed set. Even under the prevailing climatic conditions, drought induced an increase in both panicle and canopy-air temperature due to the lack of transpirational cooling [17]. Soil and plant water status, air and plant temperature, leaf and panicle transpiration rates, along with seed yield and seed quality were monitored throughout the experiments. The results showed that canopy-air temperature frequently rose above 33°C under drought stress, the temperature considered the threshold for the induction of spikelet sterility of rice in the field. This was accompanied by significant reductions in grain yield [17].

I have no concerns about the data analysis presented by the authors, only some suggestions about the best way to present it:

-Please keep font size and type uniform across figures.
Response: We thank the reviewer for pointing this out. We have revised Figures 7, 8, 10 and 11 accordingly.

-With so many venn diagrams and tables it might be useful to consider something like https://github.com/hms-dbmi/UpSetR or any other approach that can help visualize intersections between the different genotypes and treatments more easily.
Response: We are aware of these approaches but decided against using them for this paper. These visualization tools are very useful e.g. for transcriptomic data, where hundreds of genes have to be classified. However, the number of metabolites

	detectable by GC-MS is still comparatively small so that relevant metabolites in the different intersections could just be listed individually. We find this more appropriate because in this way the metabolite identities are immediately obvious to the reader, while with an approach such as UpSetR the reader would need to refer to an additional supplemental table to see the identity of the metabolites.<br><br>- Related to the point above, it would be useful to combine tables and ven diagrams that referr to the same dataset. E.g. Figure 9 and 10.<br>Response: We have combined Venn diagrams and heatmaps/tables as far as possible. However, in the case of Figures 6 and 7, and 9 and 10 the combination would require to reduce the size of the single panels to a degree that they would no longer be readable. We would therefore strongly prefer to leave these figures unaltered.<br><br>Reviewer #2: The authors conducted metabolome analysis of 3 rice cultivars with different drought tolerance. The samples are flag leaves, flowering spikelets, and developing grains collected from rice plants grown in the paddy field under combined drought and heat stress conditions. The sampling was conducted over 3 consecutive years. Based on the datasets acquired by GC-MS from these samples, the authors analyzed the metabolic responses of respective organs, and found several metabolic markers linked to the stress tolerance, yield and grain quality. Although it remains still unknown whether these marker metabolites provide drought tolerance to plants, these markers, especially deduce from the analysis of samples from plants grown under well-irrigated control conditions, might be useful for breeding of rice. Although in this experiment quite different cultivars are used (aus, indica), further analysis using more cultivars selected by considering their genetic relationship<br>might reveal more suited markers for breeding.
**Additional Information:**	
**Question**	**Response**
Are you submitting this manuscript to a special series or article collection?	No
**Experimental design and statistics**<br><br>Full details of the experimental design and statistical methods used should be given in the Methods section, as detailed in our Minimum Standards Reporting Checklist. Information essential to interpreting the data presented should be made available in the figure legends.<br><br>Have you included all the information requested in your manuscript?	Yes
**Resources**<br><br>A description of all resources used, including antibodies, cell lines, animals and software tools, with enough information to allow them to be uniquely identified, should be included in the Methods section. Authors are strongly	Yes

encouraged to cite Research Resource Identifiers (RRIDs) for antibodies, model organisms and tools, where possible.<br><br>Have you included the information requested as detailed in our Minimum Standards Reporting Checklist?	
**Availability of data and materials**<br><br>All datasets and code on which the conclusions of the paper rely must be either included in your submission or deposited in publicly available repositories (where available and ethically appropriate), referencing such data using a unique identifier in the references and in the “Availability of Data and Materials” section of your manuscript.<br><br>Have you have met the above requirement as detailed in our Minimum Standards Reporting Checklist?	Yes

Powered by Editorial Manager® and ProduXion Manager® from Aries Systems Corporation

Manuscript

Click here to view linked References

Click here to access/download;Manuscript;Lawas_et_al_GigaScience_revised

Metabolic responses of rice cultivars with different tolerance to combined drought and heat stress under field conditions

Lovely Mae F. Lawas[1], Xia Li[1,2], Alexander Erban[1], Joachim Kopka[1], S.V. Krishna Jagadish[3,4], Ellen Zuther[1], Dirk K. Hincha[1,*]

[1]Max-Planck-Institute of Molecular Plant Physiology, D-14476 Potsdam, Germany

[2]Present address: Institute of Subtropical Agriculture, Chinese Academy of Science, Changsha, Hunan, 410125, China

[3]International Rice Research Institute, DAPO Box 7777, Metro Manila, Philippines

[4]Department of Agronomy, Kansas State University, Manhattan, Kansas 66506, USA

[*]Corresponding author:

Dirk K. Hincha, Max-Planck-Institute of Molecular Plant Physiology, Am Mühlenberg 1, D-14476 Potsdam, Germany

e-mail addresses:

LMFL: Lawas@mpimp-golm.mpg.de

XL: rainbowleelx@hotmail.com

AE: Erban@mpimp-golm.mpg.de

JK: Kopka@mpimp-golm.mpg.de

SVKJ: kjagadish@ksu.edu

EZ: Zuther@mpimp-golm.mpg.de

DKH: Hincha@mpimp-golm.mpg.de

Abstract

Background: Rice is susceptible to both drought and heat stress, in particular during flowering and grain filling, when both grain yield and quality may be severely compromised. However, under field conditions, these two stresses rarely occur separately. Under well-watered conditions, plants avoid heat stress by transpirational cooling, while this is not possible under drought conditions. Although investigating combined drought and heat stress is clearly more agronomically relevant than analyzing the effects of the single stresses, only a few studies of this stress combination, in particular under field conditions, have been published.

Results: Three rice cultivars differing in drought and heat tolerance were grown in the field under control and drought conditions in three consecutive years. Drought was applied either during flowering or during early grain filling, and resulted in simultaneous heat stress, leading to reduced grain yield and quality. Analysis by gas chromatography-mass spectrometry (GC-MS) showed distinct metabolic profiles for the three investigated organs (flag leaves, flowering spikelets, developing seeds). The metabolic stress responses of the plants also strongly differed between cultivars and organs. Correlation analysis identified potential metabolic markers for grain yield and quality under combined drought and heat stress from both stress-regulated metabolites and from metabolites with constitutive differences between the cultivars.

Conclusions: GC-MS resolved metabolic responses to combined drought and heat stress in different organs of field-grown rice. The metabolite profiles can be used to identify potential marker metabolites for yield stability and grain quality that are expected to improve breeding efforts towards climate change resilient rice.

Keywords: combined stress, drought stress, flowering, grain filling, heat stress, marker metabolites, metabolomics, rice (*Oryza sativa*)

Background

Changes in air temperature and precipitation have affected the global climatic scenario, wherein global surface temperature has increased by an average of 0.85 °C during the past century while changes in precipitation varied geographically [1]. Climate models predict that heat waves and increased frequency and duration of dry conditions will persist in the future. Together with other changes in the climate system, drought and heat are expected to further negatively impact crop production [1]. Current climate changes, with emphasis on variation in temperature and precipitation, already account for 32-39% of the observed variability in the yield of major crops, including rice [2]. Rice is susceptible to heat [3–6] and drought [7–10] especially during the flowering and grain-filling stages, resulting in reduced grain yield and quality.

However, under natural field conditions, a combination of two or more stresses is more prevalent and more damaging to plants than exposure to a single stress [11]. Moreover, combined stress elicits unique responses that cannot be extrapolated from each of the individual stress responses [12,13]. The effects of combined drought and heat stress have been studied in model plants and crops at the agronomic [14–17], physiological [18–20], molecular [12,21,22], and metabolic [13,23,24] levels, encompassing different developmental stages. These studies have also shown cultivar-specific responses, and led to the identification of cultivars with superior tolerance to combined drought and heat stress. Knowledge about the molecular mechanisms underlying tolerance to this stress combination coinciding with the particularly sensitive flowering and grain-filling stages under field conditions is limited, especially in cereals

[25]. Although rice cultivation regions that are vulnerable to both drought and heat conditions have been identified [26], only a few reports on combined drought and heat stress are available [17,27–29]. With the exception of the most recent study [17], all were conducted in controlled environments where conditions are less complex compared with natural field conditions [11], which have been experimentally shown to induce more variable responses [30,31]. To date, there are no reports available that evaluate the metabolic responses to combined drought and heat stress at the flowering and grain-filling stages in field-grown rice. In addition, there is only one publication that reports such metabolic responses in different rice floral tissues, albeit from controlled environment experiments [29].

Hence, we conducted a three-year field experiment [17] and assessed the metabolic changes in both source and sink organs in response to combined drought and heat stress during flowering and early grain filling. This study specifically aimed to (i) define the metabolic profiles of flag leaves, flowering spikelets, and developing seeds under control conditions and differentiate rice cultivars with contrasting tolerance to combined drought and heat stress based on their organ-specific constitutive metabolic profiles; (ii) quantify organ- and cultivar-specific metabolic changes under stress during flowering and early grain filling compared with control conditions; (iii) compare the metabolic responses under mild and severe stress during flowering; (iv) associate constitutive and stress-responsive metabolites with stress-induced changes in grain yield and quality to identify potential metabolic stress tolerance markers.

Data description

We performed three field experiments in 2013, 2014 and 2015 at the International Rice Research Institute (IRRI) in the Philippines during the dry season (late April to early May, the

hottest time of the year) including the three rice cultivars N22, Dular and Anjali that differ in their response to drought and heat stress. Plants were either grown in flooded paddies throughout their life cycle, or were drought stressed either during flowering or early grain filling. At the end of the stress period, plants were rewatered to allow seed set. Even under the prevailing climatic conditions, drought induced an increase in both panicle and canopy-air temperature due to the lack of transpirational cooling [17]. Soil and plant water status, air and plant temperature, leaf and panicle transpiration rates, along with seed yield and seed quality were monitored throughout the experiments. The results showed that canopy-air temperature frequently rose above 33°C under drought stress, the temperature considered the threshold for the induction of spikelet sterility of rice in the field. This was accompanied by significant reductions in grain yield [17]. Over 1200 samples were taken from flag leaves, flowering spikelets and developing seeds and were analyzed for soluble metabolites by gas chromatography coupled to mass spectrometry (GC-MS). All data have been deposited in the MetaboLights database and are freely accessible. Here, we present an analysis of the metabolome data from samples obtained under well-watered control conditions, during the early, mild stress phase and during the late, more severe stress phase. According to their divergent tolerance properties, the cultivars differed in their metabolic reaction to combined drought and heat stress and also in their metabolome profiles under non-stressed conditions. We identified metabolites that were correlated in their abundance with either the reduction in yield or the loss of grain quality under stress. These metabolites constitute a starting point for the development of metabolic markers to speed up the generation of new stress-tolerant rice cultivars.

Analysis

Initial data processing

In field experiments conducted over three consecutive years, a total of 415 (2013), 406 (2014), and 420 (2015) samples were collected from flag leaves, flowering spikelets and developing seeds of three rice cultivars differing in their drought and heat response. Samples were taken from control plants (fully-flooded), during exposure to combined drought and heat stress, and during subsequent rewatering, with stress exposure either during flowering or early grain filling (Fig. 1). Agronomic and physiological analyses, as well as climate and microclimate data from these experiments, have been published recently [17]. All samples were analyzed by GC-MS metabolite profiling. A total of 221, 143, and 229 metabolites were detected in the samples from each year (Table 1), excluding contaminants that were detected by non-sample controls and added internal standards. Due to the high complexity of the obtained data sets, we will in the following present an analysis of the effects of combined drought and heat stress on the metabolome of the three investigated rice cultivars in three different organs. The effects of rewatering on the metabolome of the same cultivars and organs will be presented in a separate study. However, data pre-processing and normalization were performed for the complete data set to allow direct comparisons of this and future analyses of this large multi-factorial experiment.

PCA performed on the control and stress subset of the data (135 samples from flag leaves during the flowering stage, 90 samples from flag leaves during the early grain filling stage, 132 samples from flowering spikelets, 87 samples from developing grains) showed a clear separation between the three organs that was dominant compared to cultivar and treatment effects (Fig. 2). Source (flag leaves) and sink (flowering spikelets and developing seeds) organs were separated by PC1 that explained 48% of the total variance in the data set, while the two sink organs were separated by PC2, explaining a further 26% of the variance. Because of this strong organ

specificity of metabolite composition, we decided to perform all further data processing and analysis separately for the different organs.

Table 1. Number of analyzed samples and detected and analyzed compounds

	**Flag leaves**			**Flowering spikelets**			**Developing seeds**		
	2013	***2014***	***2015***	***2013***	***2014***	***2015***	***2013***	***2014***	***2015***
Samples analyzed by GC-MS	207	210	210	88	84	90	120	112	120
Compounds detected by GC-MS	255	164	264	255	164	264	255	164	264
Metabolites after removing contaminants and standards	221	143	229	221	143	229	221	143	229
Metabolites with ≥33.33% non-missing values	177	101	206	181	112	194	163	86	177
Metabolites common in the three experiments		81			88			67	

Note: Metabolites were identified and quantified relative to internal standards and sample fresh weight. The table reports the numbers of samples analyzed, the metabolites retained after data pre-processing, and common metabolites among the three experiments (2013, 2014, 2015). Data from flag leaves, flowering spikelets, and developing seeds were analyzed separately. The total number of detected compounds and the number of metabolites after removing contaminants and standards is expressed per experiment, hence the same number is shown for all organs within the same year. Missing values, however, were organ-specific.

Metabolites that were not detected in two-thirds or more of the samples from each organ per year were excluded from further analysis. The threshold of 33.33% detection allows for metabolites that may be present only in one of the three cultivars to be included in the analysis to capture cultivar-specific responses. This filtering resulted in the removal of 10% to 40% of the detected

metabolites per organ and year (Table 1). From the retained metabolites, we considered only those that were common across the three experiments in each organ-specific data set for detailed statistical analyses. This allowed us to use of the samples from all three experiments as replicates. Finally, 81 metabolites were used in the analysis of data from flag leaves, 88 metabolites for flowering spikelets, and 67 metabolites for developing seeds (Table 1). Additional file 1 lists all metabolites included in our analysis, separately for those only present in one organ, or in two organs or commen among all three organs.

Constitutive metabolic differences among cultivars

Metabolite profiles of all three organs significantly varied among the three contrasting cultivars (N22, drought and heat tolerant; Dular, drought tolerant, but heat sensitive; Anjali, drought and heat sensitive) already under fully flooded control conditions (Fig. 3, 4, 5). In flag leaves, more metabolites showed constitutively different levels between cultivars during flowering (47 metabolites) than early grain filling (33 metabolites) (Fig. 3). Twenty-four metabolites had significantly different constitutive levels in flag leaves between at least two of the cultivars regardless of the developmental stage of the plants. The magnitude of the differences between the cultivars was quite similar in both stages for most of these metabolites, which were mainly organic acids, sugars and sugar alcohols, such as pyruvic acid, glyceric acid-3-phosphate, erythronic acid, *myo*-inositol, and raffinose.

At the flowering stage, the levels of more than half (58%) of the identified flag leaf metabolites were significantly different between at least two cultivars. Most had the highest levels in the susceptible cultivar Anjali, as indicated by the negative $\log_2$ ratios in Fig. 3B, while mainly sugars and sugar alcohols showed the highest levels in the most tolerant cultivar N22. On

the other hand, less than half (41%) of the 81 metabolites analyzed in flag leaves significantly varied in their constitutive levels between cultivars at the early grain-filling stage. At this developmental stage, N22 and Anjali had higher levels of the majority of these metabolites than Dular (Fig. 3D). In particular, the levels of several acids, polyhydroxy acids and phosphates were higher in the most sensitive cultivar Anjali than in either N22 or Dular, while arabitol, arabinose and kestose showed the highest levels in N22.

In flowering spikelets, 64 out of 88 analyzed metabolites showed significantly different constitutive levels between at least two of the cultivars (Fig. 4). Half of these metabolites were amino acids and yet non-identified metabolites. The drought and heat tolerant N22 had the highest levels of the polyols glycerol and *myo*-inositol, and together with the equally drought tolerant Dular had higher levels of raffinose and 2-oxo-glutaric acid than the drought and heat susceptible Anjali (Fig. 4B). On the other hand, Anjali had, in addition to some amino acids, higher levels of the polyamines putrescine and spermidine and their biosynthetic precursor ornithine than Dular and N22.

Around two-thirds of the metabolites (44 out of 67) analyzed in developing seeds significantly differed in constitutive levels between cultivars (Fig. 5). In contrast to flag leaves and flowering spikelets, where Anjali exhibited constitutively higher levels of several metabolites in comparison with N22 and Dular, in developing seeds only *myo*-inositol and erythritol had significantly higher constitutive levels in Anjali compared with Dular and N22, respectively (Fig. 5B). N22 had higher levels of putrescine and two unknown metabolites (A159003 and A174001) than both other cultivars, and in addition several sugars and amino acids showed higher levels in N22 than Anjali. Dular on the other hand exhibited the highest

constitutive levels of e.g. 4-hydroxy-benzoic acid, vanillic acid, arabitol, hydroquinone and arbutin (4-hydroxyphenyl-β-glucopyranoside).

Organ-specific responses to combined drought and heat stress

Flag leaves

Some metabolic responses of flag leaves to stress during flowering (Fig. 6) differed depending on the stress duration (Additional file 2) and therefore also on stress intensity [17]. For example, only the drought and heat stress-tolerant N22 showed increased levels of an unknown metabolite and reduced levels of succinic acid and Glc-6-P under mild stress, while the same response was observed in all three cultivars under severe stress (Fig. 7, highlighted in purple). Conversely, the sensitive cultivar Anjali responded earlier than N22 in altering the levels of seven metabolites under stress (Fig. 7, highlighted in blue).

Under mild stress at the flowering stage, flag leaves of the drought-tolerant cultivars N22 and Dular showed a specific overlap of six metabolites whose levels significantly increased compared with well-watered control plants (Fig. 6A). These included Glu, putrescine, raffinose and Suc, while there were no common decreased metabolites (Fig. 7). In contrast, the overlap exclusively between N22 and Anjali yielded only one unidentified metabolite and the specific overlap between Dular and Anjali consisted of only three metabolites. On the other hand, of the 17 metabolites that showed reduced levels under combined stress, 13 were found in Anjali, of which six were in common with Dular, but only one with N22 (Fig. 6B). Under longer and more severe stress, more metabolites responded compared with mild stress (Fig. 6C and D). Most of

these metabolites were either common to all three cultivars (21 metabolites) or specific to both Dular and Anjali (16 metabolites). Of the metabolites whose levels significantly changed under mild or severe stress, approximately 20% and 33% were unique to one of the treatments (Fig. 7, highlighted in yellow and green, respectively). The levels of the remaining metabolites were significantly changed regardless of stress duration and intensity. Examples of cultivar-specific responses are the increase in the levels of Glu in N22 and the decrease of phosphoenolpyruvic acid levels in Dular and Anjali. On the other hand, an increase in the levels of Phe, Pro, Thr, and erythritol and a decrease in malic acid levels were observed in all cultivars and can thus be regarded as a general metabolic response of the flag leaves of all cultivars to both mild and severe stress during flowering (Fig. 7).

Metabolites that had significantly changed levels under severe stress during early grain filling (Fig. 6E and F), except vanillic acid, ascorbic acid, Suc, and four unknown metabolites, were also responsive to severe stress during flowering (Fig. 8). Most of these common metabolites were regulated by the same cultivar/s across both developmental stages. For instance, lower levels of phosphoric acid and malic acid under stress relative to control conditions were detected in N22 across both flowering and early grain filling. Nine metabolites had common responses in the flag leaves of all three cultivars and in both developmental stages and could thus be considered as metabolites generally responsive to severe drought and heat stress. These included the amino acids Phe, Pro, and Thr, the TCA cycle intermediates citric acid, isocitric acid, and succinic acid and raffinose.

Flowering spikelets

Similar to flag leaves during the flowering stage, combined drought and heat stress elicited more stress-responsive metabolites, in particular with increased abundance, under severe stress compared with mild stress in flowering spikelets (Fig. 9; Additional file 3). The number of metabolites that showed a significant increase or decrease during mild stress relative to control conditions was very similar. Overlap between the cultivars was small, with a maximum of four metabolites in common between the heat susceptible cultivars Dular and Anjali (Fig. 9A and B). Furthermore, the three cultivars had no common metabolites responsive to mild stress.

Longer and more severe stress induced more significant increases in metabolite levels relative to control plants, while the number of metabolites with reduced levels remained low (Fig. 9C and D). The overlap among all three cultivars consisted of 10 metabolites, comprising amino acids, polyols, and unidentified metabolites (Fig. 10). There was only little overlap between N22 and either of the other two cultivars. In contrast, 14 metabolites showed changes in content that were common exclusively between Dular and Anjali, including increased levels of Fru and Glc and reduced levels of arbutin.

While half of the metabolites whose levels were significantly changed during severe stress were unique to this time point, 24% of the metabolites responsive to mild stress were exclusive to this treatment (Fig. 10). Most metabolites common between mild and severe stress were cultivar specific. Three of these common stress-responsive metabolites were differentially regulated among the cultivars. Erythritol, Ile, and an unknown metabolite showed significantly altered levels under severe stress in all cultivars, but showed significant changes under mild stress only in the susceptible cultivars Dular and Anjali. Similarly, 1-kestose was accumulated in Dular under mild and in Anjali under severe stress.

Developing seeds

Combined drought and heat stress-induced changes in developing seeds comprised mostly of the accumulation rather than the reduction of metabolites (Fig. 11). As a general stress response of all three cultivars, the amino acids 3-cyano-Ala, Ile, and Phe and two unknown metabolites had increased, while succinic acid, Glc-6-P, erythronic acid, and erythritol had decreased levels (Fig. 11C). Otherwise, the overlap between cultivars was small. The drought-tolerant cultivars N22 and Dular shared no metabolites with the same response, whereas Anjali had two overlapping increased, but no decreased metabolites in common with N22 and Dular, respectively. Among the eight metabolites with significantly reduced levels under stress, half were unique to N22, while the other half was common to all three cultivars.

Since seeds develop from spikelets that underwent successful fertilization, it is interesting to compare the responses at these two developmental stages. Eleven out of the 28 metabolites that exhibited significant changes in levels during stress in developing seeds were unique to this organ (Fig. 11C, highlighted in green), while among those metabolites that were also stress-responsive in flowering spikelets, six exhibited the same cultivar-specific responses (Fig. 11C, highlighted in italics). For instance, arabitol accumulated in N22 under severe stress in both flowering spikelets and developing seeds. In addition, Ile also showed the same response across the two organs, but this was a general rather than a cultivar-specific response. The other metabolites showed the same pattern of change in the two organs, but in different cultivars, or exhibited an opposite response between the two organs. An example of the latter case is erythritol, which accumulated in flowering spikelets under stress in all cultivars but had reduced levels in stressed developing seeds.

Metabolite-yield correlations

Correlations between changes in metabolite levels and yield reduction under stress

From the same experiments that were used to obtain the samples for metabolite profiling, we also determined seed yield and seed quality (chalkiness) [17]. Both the extent of the reduction in the amount and quality of the harvested seeds under stress can be used as measures of the stress tolerance of the cultivars. To identify candidates for potential marker metabolites for rice tolerance to combined drought and heat stress, we performed correlation analyses between the magnitude of changes (i.e. absolute increase or decrease) in metabolite levels and yield reduction under stress relative to control conditions (Fig. 12A to F). In this context, a positive correlation means that larger changes in metabolite levels under stress indicate a smaller yield reduction, i.e. higher stress tolerance, while a negative correlation means that a larger change in metabolite levels under stress indicates higher yield reduction, i.e. lower stress tolerance. It is obvious from Fig. 12A to F that most of the identified significant correlations point to metabolites whose levels changed more strongly with higher yield reduction under stress. Only eight out of the 35 identified metabolites showed larger changes with higher stress tolerance. In addition, with the exception of isocitric acid, phosphoric acid and A159003, no metabolite showed an opposite direction of the correlation in different organs or under mild and severe stress conditions. However, one unknown metabolite (A124002) showed a negative correlation, i.e. larger changes with lower tolerance, in flowering spikelets during both mild and severe stress (Fig. 12D and E) and erythritol showed a negative correlation in both flowering spikelets and developing seeds under severe stress (Fig. 12E and F). Four additional metabolites (A174001, 3-cyano-Ala, gluconic and threonic acid) showed negative correlations in flag leaves during severe stress at the

flowering and grain-filling stage (Fig. 12B and C). All other metabolites only showed a correlation in one specific organ under one stress condition.

The largest number of significant correlations between the magnitude of change in metabolite levels and grain yield under stress was observed in flag leaves with 10 and eight metabolites during flowering stage under mild (Fig. 12A) and severe stress conditions (Fig. 12B), respectively, and with 14 metabolites under severe stress at the early grain-filling stage (Fig. 12C). There were also 14 metabolites identified in flowering spikelets under severe stress (Fig. 12E), but only five and two in developing seeds under the same stress conditions (Fig. 12F) and in flowering spikelets under mild stress (Fig. 12D), respectively. Most of the identified metabolites were unique to either source or sink organs at a certain stress condition and developmental stage, except for gluconic and threonic acid that were common in flag leaves at the flowering stage and in flowering spikelets (Fig. 12B and E), and isocitric acid which was common in flag leaves at the early grain-filling stage and in developing seeds (Fig.12C and F).

Correlations between metabolite levels under control conditions and yield reduction under stress

From a breeding perspective, metabolite markers that could be used under control conditions would be an optimal tool, as this would overcome the necessity for costly stress experiments. Therefore, we also tested the correlation of stress-induced yield loss with constitutive metabolite levels under control conditions (Fig. 12G to J). In this analysis, a positive correlation indicates that higher metabolite levels are related to a smaller yield loss under stress conditions, i.e. higher tolerance, while a negative correlation indicates that lower metabolite levels are related to higher tolerance. It is quite striking that much fewer significant correlations could be identified (11 compared to 35; compare Fig. 12G to J with Fig. 12A to F) and that most of these correlations (8

out of 11) were positive, i.e. the identified metabolites were present in higher amounts when the stress-induced yield loss was smaller. In addition, there was no overlap among the identified metabolites in different organs or at different growth stages.

Most of the significant correlations were again identified in flag leaves (8 out of 11). Five of these metabolites (arabitol, Glc-6-P, and A174001 at the flowering stage (Fig. 12G) and succinic and threonic acid at the grain-filling stage (Fig. 12H)) were also identified in the previous correlation analysis in flag leaves at the same developmental stage (metabolites indicated in red font in Fig. 12).

Only one metabolite (A214003) in flowering spikelets showed a significant positive correlation (Fig. 12I) between its levels under control conditions and yield loss under stress. On the other hand, two metabolites from developing seeds, glycerol and A155004, had significant negative correlations (Fig. 12J). None of these metabolites in the sink organs were identified in the previous correlation analysis.

Metabolite-grain quality correlations

Correlations between changes in metabolite levels and changes in the proportion of chalky grains under stress

In addition to grain yield, grain quality is also of obvious importance in a staple food crop such as rice. Grain chalkiness is a commonly used quantitative measure to describe grain quality, with an increase in chalkiness, as it occurred under the applied stress conditions [17], indicating lower quality. Rice grains with >50% chalk content are generally considered to be undesirable in the consumer market [32,33]. We therefore tested whether there were significant correlations

between changes in metabolite levels and changes in the proportion of grains with >50% chalk [17], with the aim to identify potential metabolic markers for this important trait.

A total of 26 metabolites showed a significant correlation between the magnitude of the difference in content between stress and control conditions and the change in the proportion of chalky grains (Fig. 13A to E). Except for ascorbic acid, all metabolites showed a positive correlation, indicating that the respective metabolites had larger changes in content when the proportion of chalky grains increased more strongly, i.e. with lower combined drought and heat tolerance. In contrast to the corresponding correlations with yield reduction, there were fewer correlations found in flag leaves (8) than in flowering spikelets exposed to mild (11) and severe stress (10). Surprisingly, there were no significant correlations between changes in metabolite levels and reduction in seed quality under stress in developing seeds. While there was no overlap between metabolites identified from source and sink organs, three of the metabolites showing significant correlations in flowering spikelets were identical between plants exposed to mild and severe stress (butyro-1,4-lactam, 1-kestose, and succinic acid).

Correlation between metabolite levels under control conditions and changes in the proportion of chalky grains under stress

Analogous to the approach described above for identifying constitutive metabolic markers for reduced grain yield under stress, we also investigated the relationship between the constitutive levels of metabolites and changes in the proportion of chalky grains under stress. A total of 18 metabolites with significant correlations were identified in this analysis, of which eight were found in flag leaves, seven in flowering spikelets and three in developing seeds (Fig. 13F to I). Most correlations (13 of 18) were negative, i.e. higher constitutive metabolite levels

correlated with lower fractions of chalky grains. An interesting exception are the three metabolites (A237001, A237002, and ribitol) identified from developing seeds, which all showed the opposite behavior (Fig. 13I). Furthermore, of the 18 metabolites identified as significant by this correlation analysis, seven (one in flag leaves and six in flowering spikelets) were also identified in the previous analysis as showing a significant correlation between their change in content under stress and the increase in chalky grains.

Discussion

While most investigations of stress effects on plants have focused on a single stress factor, conditions in the field often result in the simultaneous imposition of two or even more stresses. Our current knowledge suggests that the molecular and metabolic consequences of a combined stress exposure cannot be extrapolated from the effects of the single stresses (see [25] for a recent review). A common pair of companion stresses is drought and heat. For example, a recent transcriptomic study of drought effects on potato in the field revealed a massive induction of heat shock genes, indicating the inevitable induction of heat stress during drought, even under the relatively mild climatic conditions of Central Europe [31]. The experiments conducted in the present study were explicitly set up during the hottest time of the year in the Philippines, with the aim to induce heat stress as a result of the imposition of drought stress. We have presented physiological and agronomic evidence recently [17] that under these conditions drought stress resulted in a significant increase in both flag leaf and panicle temperature, accompanied by decreased grain yield and increased chalkiness of the harvested grains. It should be mentioned at this point that due to the nature of such field experiments it was not possible to generate "true controls", i.e. samples harvested under conditions where well-watered and drought-stressed

plants were exposed to a lower temperature that would not elicit heat stress. This would require growing plants during a cooler season or in a cooler region, which would obviously change too many other growth and environmental conditions to make this a relevant direct control.

Our data showed that the three investigated organs (flag leaves, flowering spikelets and developing seeds) could be clearly separated in a PCA according to their metabolite composition, with the largest difference between sink and source organs. Due to these large differences among the organs, all further analysis was performed separately on the organ-specific data sets. A similar separation has been shown previously for leaf blade and ear/husk in maize plants [34] and flag leaves and spikelets in rice [33]. Also, different flower tissues could be separated in rice and sorghum based on their metabolite and lipid profiles, respectively [29,36].

Constitutive differences in metabolite content among cultivars in relation to drought and heat tolerance

Metabolite composition did not only differ among organs, but in addition each organ showed a cultivar-specific constitutive metabolome under fully flooded control conditions. Since the cultivars differ in their stress responses, this opens the possibility that cultivar-specific metabolic pre-adaptations may be identified. There were a total of 60 annotated metabolites that showed significant differences in content between any two cultivars across all organs, i.e. flag leaves at the flowering and grain filling stage, flowering spikelets and developing seeds. Of these metabolites only seven were identified by this analysis in all organs, including two acids, three sugar alcohols, arbutin and 1-kestose. Only for vanillic acid and arbutin the highest relative amounts were detected in Dular in all organs, while the relative content in the three cultivars differed between the organs for the other metabolites. Likewise, four metabolites differed among

cultivars only in flag leaves (irrespective of developmental stage), with phosphoric acid, dehydroascorbic acid dimer and phosphoenolpyruvate showing the highest amounts in Anjali, and 5-caffeoyl-trans-quinic acid in Dular. However, the last three of these metabolites were only detectable in flag leaves, but not in flowering spikelets or developing seeds. These data alone without any further functional analysis provide no clear basis to deduce any hypotheses for metabolic pre-adaptation towards drought or combined drought and heat tolerance in the investigated cultivars.

In the sink tissues a total of 22 out of the 60 annotated metabolites described above showed significant constitutive differences between at least one pair of cultivars. Of these metabolites, 11 were specific to flowering spikelets and three to developing seeds. The latter metabolites (isocitric acid, glycerol-3-P, galactaric acid) showed the highest relative amounts in either N22 or Dular, which are both drought tolerant, in contrast to Anjali. A possible functional role of these compounds in drought tolerance, however, is presently unclear. Of the metabolites specifically identified in flowering spikelets, four that were not detectable in flag leaves or developing seeds, showed the highest content in the most susceptible cultivar Anjali (Tyr, 4-hydroxy-trans-cinnamic acid, ornithine, spermidine). In addition to spermidine, a further polyamine, putrescine, also showed the highest content in Anjali in flowering spikelets. Both putrescine and spermidine levels further increased in flowering spikelets under stress conditions in Anjali, pointing to a possible negative role of high polyamine concentrations for the drought and/or heat tolerance of rice spikelets. Similarly, high salt sensitivity was correlated with high putrescine levels in the leaves of 18 rice cultivars under control conditions [37], while no such correlation was found under drought stress in a similar panel of cultivars [38]. Conversely, developing seeds of Anjali contained the lowest amounts of several amino acids such as Gln and

Gly under control conditions that may function as compatible solutes under drought in the more tolerant cultivars. Likewise, four sugars which are well-known compatible solutes (Glc, Fru, 1-kestose and raffinose) showed the highest constitutive levels in N22 and/or Dular in all investigated organs, with the exceptions of developing seeds, where no significant differences among the cultivars could be detected for raffinose.

To identify potential constitutive metabolite markers for combined drought and heat tolerance in rice, we used the data (reduction in grain yield, increase in fraction of chalky grains, relative constitutive metabolite content) from the three years separately to obtain nine data points (three years times three cultivars) for correlation analysis. This generated additional variability in the data, as the magnitude of the stress effects on grain yield and chalkiness in the field varied between years [17]. When we correlated the magnitude of yield reduction due to drought and heat stress with constitutive metabolite pool sizes, 11 metabolites showed a significant correlation. Interestingly, most metabolites were identified in flag leaves and not in flowering spikelets, although pollen sterility is considered the most important factor for yield reduction under conditions of combined drought and heat stress in rice [27,29].

Analogous to the approach described above we also attempted to identify potential constitutive marker metabolites for grain quality under drought and heat stress. While we identified eight potential markers from the flag leaf metabolome, only three metabolites from developing seeds (A237001, A237002 and ribitol) showed significant correlations of their constitutive pool sizes with changes in the fraction of chalky grains. These correlations were all positive, indicating that increased constitutive levels of these metabolites were related to a larger increase in the fraction of chalky grains under stress. On the other hand, almost all correlations detected for metabolites in flag leaves and spikelets were negative.

The potential markers for the stability of grain yield and quality under drought and heat stress identified from the flag leaf metabolomes may not play a direct role in these traits but may function in an indirect way, possibly by characterizing the performance of source tissues that ultimately export metabolites to flowering spikelets and developing seeds. It may be the export potential of the sources tissue for carbon and possibly nitrogen that may influence the quality and yield of seeds. Alternatively, the compounds could be merely associated to the phenotype without a functional connection, which would, however, not reduce their utility for breeding. In particular, marker metabolites that can be detected in unstressed flag leaves are interesting from a practical point of view, as they would not require stress experiments and flag leaves would be easy to sample even under field conditions.

Stress-induced changes in metabolite content among cultivars in relation to drought and heat tolerance

The metabolomic responses to drought and heat stress varied depending on the organ, cultivar and the duration and intensity of the stress treatment. Due to the fact that we investigated responses to both mild and severe stress in flag leaves at the flowering stage and in flowering spikelets, it was possible to identify metabolites in these organs that were differentially regulated with stress severity in the different cultivars. There were only a few metabolites that showed a response specifically to mild stress in the most tolerant cultivar N22 and a response to severe stress in all three cultivars, indicating metabolic changes that may be related to drought and heat tolerance. One (A155004) was increased in pool size, while two (Glc-6-P and succinic acid) were decreased. A decrease in succinic acid levels with increasing leaf temperature has previously been reported for maize leaves under drought stress [34]. Interestingly, both Glc-6-P

and succinic acid also showed decreased pool sizes in all three cultivars under severe stress in developing seeds. Both metabolites were also identified in an earlier study in the anthers of N22 as related to heat, or drought and heat tolerance, respectively [29]. In addition, the enzyme Glc-6-P dehydrogenase shows increased activity during both drought and heat stress in different plant species, leading to a reduction in Glc-6-P levels (see [39] for a review). While we did not identify any metabolites as specifically regulated in N22 in flowering spikelets under mild stress, succinic acid was also identified to have the highest constitutive content in flowering spikelets in N22. Furthermore, our analyses indicated that high constitutive levels of both compounds in flag leaves were significantly correlated with a lower yield reduction under stress. In addition, the changes in content under stress in flag leaves for both metabolites were correlated with yield reduction, and for succinic acid changes under mild stress in flowering spikelets were correlated with changes in the fraction of chalky grains.

Conversely, we also identified metabolites with specifically changed levels under mild stress in the most sensitive cultivar Anjali and a later response to severe stress in all three cultivars. The only metabolites that showed such a pattern with an early increase under mild stress in flag leaves and flowering spikelets were arabitol and Ile, respectively. Arabitol showed a significantly higher content in flag leaves at the flowering stage in N22 than in Anjali already under control conditions. The change in arabitol content under severe stress and the constitutive levels of arabitol in flag leaves were further significantly correlated with the reduction in grain yield under stress. In addition, arabitol content increased in flowering spikelets under severe stress in all cultivars and showed significant correlations with the change in chalky grain fraction both for its constitutive level and its change in content under severe stress.

Of the metabolites with specifically reduced levels under mild stress in Anjali and reduced levels in all cultivars under severe stress in flowering spikelets, pyruvic acid is of particular interest, because its constitutive content and the change in content under severe stress were significantly correlated with the change in the fraction of chalky grains after drought and heat stress. In addition, the change in pyruvic acid levels during stress in developing seeds was significantly correlated with yield reduction. Cumulatively, this evidence points to Glc-6-P, arabitol, succinic and pyruvic acid as promising metabolic marker candidates for drought and heat tolerance in rice.

We further hypothesized that metabolites whose levels under drought and heat stress were uniquely increased in the tolerant cultivar N22 are potential candidates for conferring tolerance to this stress combination in rice and may therefore be valuable targets for marker-assisted breeding. In particular, we focus here on metabolites whose levels were significantly increased under severe stress (i.e. longer stress duration and higher intensity) relative to well-watered control conditions.

In flag leaves during flowering, Glu and arbutin levels significantly increased under severe stress specifically in N22 and in flag leaves during early grain filling vanillic acid levels were significantly increased. Arbutin is an aromatic compound with strong antioxidative [40] and membrane stabilizing [41] properties. It has also been identified as a metabolic predictor of drought tolerance in potato [31,42]. Similarly, vanillic acid was identified as a drought-induced metabolite in rice leaves in the vegetative growth stage, with higher content in a drought tolerant than a sensitive cultivar [43]. In the case of flowering spikelets and developing seeds, five and one metabolite, respectively, showed a N22-specific increase under severe stress. However, the constitutive levels of these metabolites were significantly lower in N22 compared with Dular

and/or Anjali under control conditions. Therefore, even with increased levels under stress in N22, they were still lower compared with the constitutive levels of the susceptible cultivars making a functional role in drought and heat tolerance unlikely.

Potential implications

From the metabolomic analysis presented in this paper it becomes apparent that we should be cautious when comparing data from different studies that were conducted on different plant organs or tissues, or even on the same organ at different developmental stages such as flag leaves during flowering and early grain filling. Also, our study has identified a number of potential marker metabolites for both grain yield and grain quality under combined drought and heat stress. The utility of such markers has to be tested with a wider range of cultivars to assess whether more tolerant genotypes could be identified in this way. Obviously, most of the potential markers, such as those identified in flag leaves for grain quality, will most likely not have a direct function in the observed phenotype. Instead, these metabolites may indicate the status or source potential of the leaf tissue. The fact that we currently cannot functionally link systemic metabolite levels to yield reduction or changes in grain quality under stress does not diminish their potential value as markers for breeding purposes. In particular, markers that can be used without the need for stress experiments and that can be identified in plant organs such as leaves that are easy to obtain, would be of particular interest. The fact that this is already the second species after potato [42] for which predictive metabolic markers for drought and heat tolerance have been identified raises the possibility that our approach for metabolic marker discovery may be a more generally applicable strategy in efforts to breed for more stress-tolerant cultivars in different crop species. It should finally be emphasized that both studies employed field

experiments, indicating that it is indeed feasible to conduct such molecular studies under agronomically relevant conditions.

Methods

Crop husbandry and stress treatment

Crop husbandry and stress treatments were exactly the same as in Lawas et al. [17]. In brief, a three-year field experiment was conducted at the IRRI, Philippines during the dry seasons of 2013 – 2015. Rice (*Oryza sativa* L.) cultivars used were the drought, heat, and combined drought and heat tolerant N22 (*aus* ssp.), drought tolerant but heat and combined drought and heat susceptible Dular (*aus* ssp.), and drought, heat, and combined drought and heat susceptible Anjali (*indica* ssp.), which have contrasting responses to drought and heat stress at both the flowering and grain filling stages [17,27, 44–46]. These cultivars were randomly assigned in a split-plot design with three replicate subplots per treatment, with separate plots allocated for stage-specific (flowering and early grain filling) drought stress. A staggered-sowing approach was employed to ensure that the three cultivars synchronized with flowering and early grain filling that coincided with the hottest period (late April to early May) in the experiment location. The average maximum ambient air temperature coinciding with drought stress across the three years was 34.3 ± 0.50 °C (see [17] for more detailed climate and microclimate data). A rainout shelter was used for imposing drought conditions. Fully-flooded conditions were maintained until the early booting stage or until the start of flowering for flowering and early grain-filling stage drought treatments, respectively, after which water was drained from the stress plots (Fig. 1). At the end of the drought stress treatment, an average soil water potential of -46.6 ± 11.1 kPa was recorded across the three years before rewatering to maintain flooded conditions until crop

maturity. Control plots were kept fully-flooded during the entire experiment. It should be stressed that under field conditions all plants were exposed to the same environmental conditions. Hence, no "true" control at a lower air temperature was possible.

Sample collection

Three to five replicates each of flag leaves, flowering spikelets, and developing seeds per cultivar were collected under mild stress (4-7 days after drought stress initiation; Fig. 1, yellow arrow), severe stress (11-16 days after drought stress initiation; Fig. 1, red arrows), and at three rewatering time points (12, 36, and 60 h after rewaterin; Fig. 1, blue arrows g) in each of the three years, yielding a total of 12 to 15 replicate samples from each organ per treatment and time point across the three years. Mild and severe stress corresponded to an average soil water potential of -16.2 ± 4.1 kPa and -46.6 ± 11.1 kPa and an average maximum canopy air temperature in 2013 and 2014 (no data available for 2015 [17]) of 32.8 ± 0.98 °C and 35.7 ± 1.07 °C, respectively, across the experiments. For the early grain filling stage, drought stress was initiated when the plants started to flower. Hence, it was possible to collect flag leaves and spikelets at the flowering stage under mild stress conditions for comparison with the severe stress flowering samples. However, collection of early grain filling samples exposed to mild stress was not feasible as it would require a different strategy, where drought would be initiated at the start of grain filling.

When plants in the plots designated for imposing drought during early grain filling were at the flowering stage, i.e. a few days after water was drained, flowering spikelets were identified in both control and stress plots and the respective pedicels (or secondary branches in the cases where all spikelets on that branch were flowering) were marked using marker pens. Spikelets

were not directly marked on the floral tissue, i.e. lemma and palea, to avoid any interference of the chemicals from the marker pen ink with metabolite analysis. This marking strategy (only for spikelets which were collected at a later time point, i.e. during severe stress, as developing seeds) was followed to ensure that samples at the same developmental stage were harvested and that only those that were stressed during early grain filling would be collected. The marked spikelets were collected during severe stress, i.e. 9-11 days after flowering (DAF), and after rewatering, i.e. 10-14 DAF, as developing seeds. In addition, from the same plot, flowering spikelets were also collected to serve as samples exposed to mild stress during the flowering stage. On the other hand, from the flowering stage drought stress plots, spikelets flowering at the time of sampling were collected during severe stress and after rewatering. In parallel, samples from the control plots (fully-flooded, 33.3 ± 0.77 °C average maximum canopy air temperature in 2013 and 2014) were also collected in all three years. Flowering spikelets and developing seeds were individually detached from the rachis, excluding the pedicel, using forceps. Only those that were positioned at the upper two-thirds of the panicle were collected to exclude inferior spikelets, which have lower fertility compared with superior spikelets under both control and stress conditions in fully exserted panicles and in panicles partially trapped inside the flag leaf sheath [47–49]. Sampling was done randomly from at least four plants per replicate plot in order to collect at least 150 mg of tissue, with each sample replicate consisting of pooled samples from the three replicate plots. In the case of flag leaves, two leaves per replicate plot were collected randomly from plants that were at the target developmental stage. All samples were collected in 15 mL conical tubes immersed in liquid nitrogen during the entire sampling in the field and stored at -80 °C until use. Sample collection was done between 09:00 and 11:30 a.m. . in all treatments and cultivars to avoid confounding results due to the impact on circadian rhythm.

Metabolite profiling and data processing

Samples were homogenized using a cryogenic grinding robot (Labman Automation Ltd., North Yorkshire, United Kingdom). Metabolite profiling was performed as previously described [29,44]. An aliquot of 120 ± 5 mg ground tissue was used to extract a fraction enriched in polar primary metabolites and small secondary products using methanol:chloroform with $^{13}C_6$-sorbitol added as an internal standard. An aliquot of 160 µL from the upper polar phase was dried overnight in a vacuum concentrator. Chemical derivatization and gas chromatography coupled to electron impact ionization-time of flight-mass spectrometry (GC/EI-TOF-MS) was performed using a gas chromatograph with a split and splitless injector (Agilent 6890N24, Agilent Technologies, Böblingen, Germany) attached to a Pegasus III time-of-flight mass spectrometer (LECO Instrumente GmbH, Mönchengladbach, Germany) following Erban et al. [50]. Acquired chromatograms were processed by ChromaTOF software (LECO Instrumente GmbH, Mönchengladbach, Germany). Metabolites were identified using TagFinder [51], NIST mass spectral search and comparison software (NIST17; https://www.nist.gov/srd/nist-standard-reference-database-1a-v17), and the mass spectral and retention time index reference collection of the Golm Metabolome Database (GMD; http://gmd.mpimp-golm.mpg.de/). The majority of the metabolites were quantified in splitless injection mode, while malic acid, phosphoric acid, Fru, Glc, and Suc were quantified in split injection mode. Data with the best quantitative information based on manual curation were chosen and mass spectral intensity was normalized to the sample fresh weight and $^{13}C_6$-sorbitol and was used for further data analysis. Quantified metabolites were either known or yet non-identified (indicated by an identifier number) and are

archived in the Golm Metabolome Database [52,53]. All metabolomics data are freely available [54].

Statistical analysis

All statistical analyses were performed using R version 3.4.0 [55] and RStudio version 1.0.153 [56]. Data was filtered by removing all contaminants and internal standards. Principal component analysis (PCA) was performed on the data from control and stress conditions. Only metabolites identified in common from all three experiments (110 metabolites) were included in the PCA. For each metabolite, data was divided by the median across all samples and log_{10}-transformed. Mean values were pareto-scaled and mean-centered for PCA using the probabilistic method performed with the *'pcaMethods'* package (version 1.60.0), which is recommended for large data sets due to its speed and use with incomplete data sets [57]. Data visualization by score plot was done with the *'ggplot2'* package (version 2.2.1). Since the three organs (flag leaves, flowering spikelets, developing seeds) clustered distinctly from each other, further analyses were performed separately for each organ.

Each organ-specific data set was initially pre-processed separately per experiment. Although this paper focuses only on the differential metabolic responses between control and stress conditions, all data pre-processing (e.g. outlier detection, median transformation) were performed including samples collected during the rewatering time points. This will allow for a direct comparison of the data presented here and those of the metabolic differences between control and rewatering and between stress and rewatering in the future. Metabolites that were present in at least one-third of the total number of samples were considered for further data pre-processing. Hydroquinone in flowering spikelets collected in 2013, although present in only

32.95% of the total samples, was also included since it was specific to Dular based on manual inspection. Furthermore, from the reduced metabolite list, only those that were common in the three experiments were analyzed. Missing values were substituted by half of the minimum value of each metabolite for data transformation to be performed [58]. Data from the three experiments were then combined and subjected to an ANOVA-based normalization with treatment, time point, cultivar, measurement batch and sequence as factors. Systematic differences due to measurement batch and sequence were removed [59] to allow combined analysis of individual experiments [31]. The normalized data was separated into the three experiments, each of which were subjected to outlier detection using an R script [31] based on Grubbs' test from the *'outlier'* package (version 0.14). Identified outliers were replaced with missing values. Since some of the outliers were condition-specific, manual supervision of the data was also done to avoid removing biological variance. Three flowering spikelet samples that exhibited a large fraction of missing values (61-67% of metabolites not detected) at the start of data pre-processing and after outlier detection were excluded from the analysis. Moreover, data from flag leaves was separated between the flowering and early grain-filling stage after outlier detection. Data from the three experiments were then combined for each organ (flag leaves from flowering stage, flag leaves from early grain-filling stage, flowering spikelets, developing seeds) and the subsequent data processing and analyses were done on the combined data sets (12-15 replicates per organ per cultivar and condition). Data was normalized to the median of each metabolite and log_2-transformed to approximate normal distribution. Shapiro-Wilk test was performed using the R package *'stats'* (version 3.4.0) to assess normality of the data. Since not all metabolites were normally distributed, Wilcoxon-Mann-Whitney test from the R package *'stats'* was used to determine the significance of differences (expressed as log_2 fold change) of the relative

metabolite content of each cultivar under control and stress conditions. A subset of the combined data comprising only control samples was also analyzed. This data set was also median-normalized per metabolite and log_2-transformed. Wilcoxon-Mann-Whitney test (*'stats'* package) was used to compare the relative levels of metabolites between the cultivars. Venn diagrams showing unique and common metabolites between the comparisons were drawn using the *'VennDiagram'* package in R (version 1.6.17). Heat maps to illustrate metabolites with significant log_2-fold differences in content between cultivars were generated from the *'gplots'* package (version 3.0.1).

Correlations between the changes in yield and proportion of grains with >50% chalk content as a measure of grain quality under stress relative to control conditions and in the relative metabolite levels under control condition as well as the change in metabolite levels between the treatments were assessed using the Spearman's rank method from the R package *'stats'*. For ease of term, we have used "chalky grains" to refer to rice grains having >50% chalk content. Data for grain yield and proportion of chalky grains were extracted from our previous report [17]. For the correlation test, average values per cultivar per experiment (three cultivars x three years) were used. Metabolite data were median-normalized and log_2-transformed before calculating the average values per experiment. Metabolites with significant correlation between the factors of interest were visualized through heat maps (*'gplots'* package). All code used in these analyses is freely available [60].

Acknowledgements

This project has been supported by the German Federal Ministry for Economic Cooperation and Development [Project Number 11.7860.7-001.00; Contract Numbers 81141844 and 81170348]

and by the Max-Planck Society. LMFL is supported by a PhD fellowship from the University of Potsdam, Germany.

Competing interests

The authors declare that they have no financial or non-financial competing interests.

Figure legends

Figure 1. Schematic illustration of drought stress imposition and collection of samples for metabolomic analysis

Light red indicates the onset of stress and dark red indicates higher stress intensity (modified from [17]). The yellow arrow in the early grain-filling stage indicates the collection time point of flowering spikelets and flag leaves under mild stress during flowering. The red arrows indicate collection time points of flag leaves (both stages), flowering spikelets (flowering stage), and developing seeds (early grain filling stage) under severe stress, and blue arrows indicate collection time points of flag leaves (both stages), flowering spikelets (flowering stage), and developing seeds (early grain filling stage) during rewatering.

Figure 2. Score plot from the principal component analysis of rice metabolite profiles

The first two principal components (PC1 and PC2) are shown for samples from flag leaves, flowering spikelets, and developing seeds collected under control, mild stress (flag leaves at flowering stage and flowering spikelets only), and severe stress conditions from the rice cultivars N22, Dular, and Anjali. Scores are averages of the median-normalized and $\log_{10}$-transformed

mass spectral intensities (normalized to internal standard and sample fresh weight) of 110 metabolites that were detected in common across the three experiments in all organs.

Figure 3. Pair-wise comparison of metabolite levels in flag leaves of three rice cultivars under control conditions

Venn diagrams (A, C) show the number of common and specific metabolites that have significant (Mann-Whitney-Wilcoxon test, $P < 0.05$) differences in constitutive content between any pair of cultivars. The corresponding metabolites are illustrated in heat maps (B, D) with the level of significance indicated by asterisks (* $P < 0.05$; ** $P < 0.01$; *** $P < 0.001$) and the $\log_2$-fold difference indicated by the color code. Samples were taken from flag leaves under control conditions during flowering (A, B) and early grain filling (C, D). Metabolites are listed alphabetically by metabolite class, with each class identified by the depicted color code. Metabolites common between flowering and early grain filling stage are indicated in black font, while those in red font are developmental stage-specific.

Figure 4. Pair-wise comparison of metabolite levels in flowering spikelets of three rice cultivars under control conditions

Venn diagram (A) shows the number of common and specific metabolites that have significant (Mann-Whitney-Wilcoxon test, $P < 0.05$) differences in constitutive content between any pair of cultivars. The corresponding metabolites are illustrated in the heat map (B) with the level of significance indicated by asterisks (* $P < 0.05$; ** $P < 0.01$; *** $P < 0.001$) and the $\log_2$-fold difference indicated by the color code. Metabolites are listed alphabetically by metabolite class, with each class identified by the depicted color code.

Figure 5. Pair-wise comparison of metabolite levels in developing seeds of three rice cultivars under control conditions

Venn diagram (A) shows the number of common and specific metabolites that have significant (Mann-Whitney-Wilcoxon test, $P < 0.05$) differences in constitutive content between any pair of cultivars. The corresponding metabolites are illustrated in the heat map (B) with the level of significance indicated by asterisks (* $P < 0.05$; ** $P < 0.01$; *** $P < 0.001$) and the log_2-fold difference indicated by the color code. Metabolites are listed alphabetically by metabolite class, with each class identified by the depicted color code.

Figure 6. Venn diagrams illustrating numbers of metabolites with changes in abundance in flag leaves under stress

Numbers indicate common and cultivar-specific metabolites in flag leaves that showed significant (Mann-Whitney-Wilcoxon test, $P < 0.05$) increases (A, C, E) or decreases (B, D, F) in abundance during mild stress at the flowering stage (A, B), severe stress at the flowering stage (C, D), and severe stress at the early grain-filling stage (E, F) relative to well-watered controls.

Figure 7. Common and cultivar-specific stress-responsive metabolites in flag leaves during flowering

Metabolites corresponding to the numbers in the Venn diagrams in Fig. 6A to D. Comparisons between responses to early, mild and late, severe stress are color-coded: orange – common response irrespective of stress intensity; yellow – mild stress-specific; green – severe stress-specific; purple – rapid response in tolerant cultivar N22, delayed response in the more

susceptible cultivars; blue – rapid response in susceptible cultivars, delayed response in tolerant cultivar. Severe stress responses in flag leaves common between the flowering (Fig. 7) and early grain-filling stages (Fig. 8) are differentiated by font style: **bold** – common responses among all cultivars between the two stages; *italics* – cultivar-specific metabolites with similar responses in the same cultivars across the two stages. Quinic acid, 5-caffeoyl-, trans- is listed as chlorogenic acid; 4-hydroxyphenyl-β-glucopyranoside as arbutin; glyceric acid -3-phosphate as glycerate-3-phosphate; phosphoenolpyruvic acid as phosphoenolpyruvate; phosphoric acid monomethyl ester as monomethyl phosphate.

Figure 8. Common and cultivar-specific stress-responsive metabolites in flag leaves during early grain filling

Metabolites corresponding to the numbers in the Venn diagrams in Fig. 6E and F. Responses of flag leaves to severe stress common between the flowering (Fig. 7) and early grain-filling stages are indicated by font style: **bold** – common response among all cultivars between the two stages; *italics* – cultivar-specific metabolites with similar responses in the same cultivars across the two stages. Glyceric acid-3-phosphate is listed as glycerate-3-phosphate; phosphoenolpyruvic acid as phosphoenolpyruvate.

Figure 9. Venn diagrams illustrating numbers of metabolites with changes in abundance in flowering spikelets under stress

Numbers indicate common and cultivar-specific metabolites in flowering spikelets that showed significant (Mann-Whitney-Wilcoxon test, $P < 0.05$) increases (A, C) or decreases (B, D) in

abundance in response to mild (A, B) or severe stress (C, D) during the flowering stage relative to well-watered controls.

Figure 10. Common and cultivar-specific stress-responsive metabolites in flowering spikelets

Metabolites corresponding to the numbers in the Venn diagrams in Fig. 9. Comparisons between mild and severe stress are color-coded: orange – common response irrespective of stress intensity; yellow – mild stress-specific; green – severe stress-specific; blue – rapid response in susceptible cultivars, delayed response in the tolerant cultivar N22. Common responses of flowering spikelets (Fig. 10) and developing seeds (Fig. 11C) during severe stress are indicated by font style: **bold** - same response among all cultivars between the two organs; *italics* - cultivar-specific with similar responses in the same cultivars across the two organs. 4-Hydroxyphenyl-β-glucopyranoside is listed as arbutin; phosphoric acid monomethyl ester as monomethyl phosphate.

Figure 11. Common and cultivar-specific stress-responsive metabolites in developing seeds

Panels (A) and (B) show the numbers of common and cultivar-specific metabolites in developing seeds that showed significant (Mann-Whitney-Wilcoxon test, $P < 0.05$) increases (A) or decreases (B) in abundance under severe stress relative to well-watered controls. The corresponding metabolites are listed in (C). Metabolites highlighted in green showed specific responses to severe stress in developing seeds compared with flowering spikelets; metabolites in *italics* are those that have similar cultivar-specific responses across the two organs, and **bold** font

indicates metabolites with a common response with flowering spikelets among all cultivars (Fig. 10). 4-Hydroxyphenyl-β-glucopyranoside is listed as arbutin.

Figure 12. Identification of potential metabolite markers for yield stability under combined drought and heat stress

The upper panels (A – F) show metabolites with significant correlations (Spearman's rank correlation, $P < 0.05$) between yield reduction under stress and the corresponding changes in metabolite content (log_2-fold change). Metabolites were analyzed in flag leaves under mild (A) and severe (B) stress at the flowering stage, and under severe stress at the early grain-filling stage (C). Flowering spikelets were investigated under mild (D) and severe stress (E) and developing seeds (F) under severe stress. The lower panels (G – J) show metabolites with significant correlations between yield reduction under stress and the relative metabolite levels under control conditions. Metabolites were analyzed in flag leaves during flowering (G) and early grain-filling stages (H), in flowering spikelets (I), and developing seeds (J). Blue and red indicate negative and positive correlations, respectively. Metabolites in dashed box are common between mild and severe stress within the same organ. Solid boxes indicate metabolites common during severe stress between flowering and early grain filling within the same source/sink organ. Metabolites indicated in red font are common between the upper and lower panels within the same organ. Metabolites are sorted alphabetically.

Figure 13. Identification of potential metabolite markers for seed quality under combined drought and heat stress

The upper panels (A – E) show metabolites with significant correlations (Spearman's rank correlation, $P < 0.05$) between increases in the proportion of grains with >50% chalk under stress and the corresponding changes in metabolite content ($\log_2$-fold change). Metabolites were analyzed in flag leaves under mild (A) and severe (B) stress at the flowering stage, and under severe stress at the early grain-filling stage (C). Flowering spikelets were investigated under mild (D) and severe stress (E). The lower panels (F – I) show metabolites with significant correlations between the increase in proportion of grains with >50% chalk under stress and relative metabolite levels under control conditions. Metabolites were analyzed in flag leaves during flowering (F) and early grain-filling stages (G), in flowering spikelets (H), and developing seeds (I). Blue and red indicate negative and positive correlations, respectively. Metabolites in dashed box are common between mild and severe stress within the same organ. Metabolites indicated in red font are common between the upper and lower panels within the same organ. Metabolites are sorted alphabetically.

Additional files

Additional file 1 (XLS). Common and organ-specific metabolites used in the final data analysis

Metabolites that were common among the three experiments with ≥33.33% non-missing values, as indicated in Table 1 and that were either specific to one or two organs, or detected in all three organs. Metabolites are listed alpahabetically by metabolite class in the same order as in Figs. 3-5.

Additional file 2 (PDF). Metabolites with different abundance under mild and severe drought and heat stress in flag leaves

The heat map displays all flag leaf metabolites that showed a significant (Mann-Whitney-Wilcoxon test, $P < 0.05$) difference in abundance between mild and severe combined drought and heat stress during the flowering stage. The level of significance is indicated for each metabolite and cultivar by asterisks (* $P < 0.05$; ** $P < 0.01$; *** $P < 0.001$) and the $\log_2$-fold difference is indicated by the color code.

Additional file 3 (PDF). Metabolites with different abundance during mild and severe drought and heat stress in flowering spikelets

The heat map displays all metabolites in flowering spikelets that showed a significant (Mann-Whitney-Wilcoxon test, $P < 0.05$) difference in abundance between mild and severe combined drought and heat stress during the flowering stage. The level of significance is indicated for each metabolite and cultivar by asterisks (* $P < 0.05$; ** $P < 0.01$; *** $P < 0.001$) and the $\log_2$-fold difference is indicated by the color code.

Availability of supporting data and materials

The data set supporting the results of this article is available in the EMBL-EBI MetaboLights database (DOI: 10.1093/nar/gks1004. PubMed PMID: 23109552) with the identifier MTBLS801.

Availability of source code and requirements

Project name: Rice_HxD_Metabolomics

Project home page: GitHub (https://github.com/llawas/Rice_HxD_Metabolomics)

Operating system: Windows 7

Programming language: R

License: GNU General Public License

References

1. IPCC. Climate Change 2014: Synthesis Report. Contribution of Working Groups I, II and III to the Fifth Assessment Report of the Intergovernmental Panel on Climate Change. Core Writing Team, Pachauri RK, Meyer LA, editors. Geneva, Switzerland: Cambridge University Press; 2014.

2. Ray DK, Gerber JS, Macdonald GK, West PC. Climate variation explains a third of global crop yield variability. Nat Commun. 2015;6:1–9.

3. González-Schain N, Dreni L, Lawas LMF, Galbiati M, Colombo L, Heuer S, et al. Genome-wide transcriptome analysis during anthesis reveals new insights into the molecular basis of heat stress responses in tolerant and sensitive rice varieties. Plant Cell Physiol. 2016;57:57–68.

4. Jagadish SVK, Craufurd PQ, Wheeler TR. Phenotyping parents of mapping populations of rice for heat tolerance during anthesis. Crop Sci. 2008;48:1140–6.

5. Shi W, Lawas LMF, Raju BR, Jagadish SVK. Acquired thermo-tolerance and trans-generational heat stress response at flowering in rice. J Agron Crop Sci. 2016;202:309–19.

6. Fitzgerald MA, Resurreccion AP. Maintaining the yield of edible rice in a warming world. Funct Plant Biol. 2009;36:1037–45.

7. Kadam NN, Struik PC, Rebolledo MC, Yin X, Jagadish SVK. Genome-wide association reveals novel genomic loci controlling rice grain yield and its component traits under water-deficit stress during the reproductive stage. J Exp Bot. 2018;69:4017–32.

8. Kumar A, Verulkar S, Dixit S, Chauhan B, Bernier J, Venuprasad R, et al. Yield and yield-attributing traits of rice (*Oryza sativa* L.) under lowland drought and suitability of early vigor as a selection criterion. Field Crop Res. 2009;114:99–107.

9. Torres RO, McNally KL, Cruz CV, Serraj R, Henry A. Screening of rice Genebank germplasm for yield and selection of new drought tolerance donors. Field Crop Res. 2013;147:12–22.

10. Pantuwan G, Fukai S, Cooper M, Rajataserreekul S, O'Toole JC. Yield response of rice (*Oryza sativa* L.) genotypes to different types of drought under rainfed lowlands Part 1. Grain yield and yield components. Field Crop Res. 2002;73:153–68.

11. Suzuki N, Rivero RM, Shulaev V, Blumwald E, Mittler R. Abiotic and biotic stress combinations. New Phytol. 2014;203:32–43.

12. Rizhsky L, Liang H, Mittler R. The combined effect of drought stress and heat shock on gene expression in tobacco. Plant Physiol. 2002;130:1143–51.

13. Rizhsky L, Liang H, Shuman J, Shulaev V, Davletova S, Mittler R. When defense pathways collide. The response of Arabidopsis to a combination of drought and heat stress. Plant Physiol. 2004;134:1683–96.

14. Cairns JE, Crossa J, Zaidi PH, Grudloyma P, Sanchez C, Araus JL, et al. Identification of drought, heat, and combined drought and heat tolerant donors in maize. Crop Sci. 2013;53:1335.

15. Mahrookashani A, Siebert S, Hüging H, Ewert F. Independent and combined effects of high temperature and drought stress around anthesis on wheat. J Agron Crop Sci. 2017;203:453–63.

16. Trachsel S, Leyva M, Lopez M, Suarez EA, Mendoza A, Montiel NG, et al. Identification of tropical maize germplasm with tolerance to drought, nitrogen deficiency, and combined heat and drought stresses. Crop Sci. 2016;56:3031–45.

17. Lawas LMF, Shi W, Yoshimoto M, Hasegawa T, Hincha DK, Zuther E, et al. Combined drought and heat stress impact during flowering and grain filling in contrasting rice cultivars grown under field conditions. Field Crop Res. 2018;229:66–77.

18. Mahalingam R. Phenotypic, physiological and malt quality analyses of US barley varieties subjected to short periods of heat and drought stress. J Cereal Sci. 2017;76:199–205.

19. Pradhan GP, Prasad PVV, Fritz AK, Kirkham MB, Gill BS. Effects of drought and high temperature stress on synthetic hexaploid wheat. Funct Plant Biol. 2012;39:190–8.

20. Perdomo JA, Conesa M, Medrano H, Ribas-Carbó M, Galmés J. Effects of long-term individual and combined water and temperature stress on the growth of rice, wheat and maize: Relationship with morphological and physiological acclimation. Physiol Plant. 2015;155:149–65.

21. Shaar-Moshe L, Blumwald E, Peleg Z. Unique physiological and transcriptional shifts under combinations of salinity, drought, and heat. Plant Physiol. 2017;174:421–34.

22. Zhao F, Zhang D, Zhao Y, Wang W, Yang H, Tai F, et al. The difference of physiological and proteomic changes in maize leaves adaptation to drought, heat, and combined both stresses. Front Plant Sci. 2016;7:1471.

23. Obata T, Witt S, Lisec J, Palacios-Rojas N, Florez-Sarasa I, Araus JL, et al. Metabolite profiles of maize leaves in drought, heat and combined stress field trials reveal the relationship between metabolism and grain yield. Plant Physiol. 2015;169:2665–83.

24. Templer SE, Ammon A, Pscheidt D, Ciobotea O, Schuy C, McCollum C, et al. Metabolite profiling of barley flag leaves under drought and combined heat and drought stress reveals metabolic QTLs for metabolites associated with antioxidant defense. J Exp Bot. 2017;68:1697–713.

25. Lawas LMF, Zuther E, Jagadish SVK, Hincha DK. Molecular mechanisms of combined heat and drought stress resilience in cereals. Curr Opin Plant Biol. 2018;45:212–7.

26. Wassmann R, Jagadish SVK, Sumfleth K, Pathak H, Howell G, Ismail A, et al. Regional Vulnerability of Climate Change Impacts on Asian Rice Production and Scope for Adaptation. In: Sparks DL, editor. Adv Agron. Burlington: Academic Press; 2009. p. 91–133.

27. Rang ZW, Jagadish SVK, Zhou QM, Craufurd PQ, Heuer S. Effect of high temperature and water stress on pollen germination and spikelet fertility in rice. Environ Exp Bot. 2011;70:58–65.

28. Jagadish SVK, Muthurajan R, Rang ZW, Malo R, Heuer S, Bennett J, et al. Spikelet proteomic response to combined water deficit and heat stress in rice (*Oryza sativa* cv. N22). Rice. 2011;4:1–11.

29. Li X, Lawas LMF, Malo R, Glaubitz U, Erban A, Mauleon R, et al. Metabolic and transcriptomic signatures of rice floral organs reveal sugar starvation as a factor in reproductive failure under heat and drought stress. Plant, Cell Environ. 2015;38:2171–92.

30. Bahuguna RN, Jha J, Pal M, Shah D, Lawas LM, Khetarpal S, et al. Physiological and biochemical characterization of NERICA-L-44: A novel source of heat tolerance at the vegetative and reproductive stages in rice. Physiol Plant. 2015;154:543–59.

31. Sprenger H, Kurowsky C, Horn R, Erban A, Seddig S, Rudack K, et al. The drought response of potato reference cultivars with contrasting tolerance. Plant Cell Environ. 2016;39:2370–89.

32. Lanning SB, Siebenmorgen TJ, Counce PA, Ambardekar AA, Mauromoustakos A. Extreme nighttime air temperatures in 2010 impact rice chalkiness and milling quality. Field Crop Res. 2011;124:132–6.

33. Lyman NB, Jagadish KSV, Nalley LL, Dixon BL, Siebenmorgen T. Neglecting rice milling yield and quality underestimates economic losses from high-temperature stress. PLoS ONE. 2013;8:e72157.

34. Witt S, Galicia L, Lisec J, Cairns J, Tiessen A, Araus JL, et al. Metabolic and phenotypic responses of greenhouse-grown maize hybrids to experimentally controlled drought stress. Mol Plant. 2012;5:401–17.

35. Raorane ML, Pabuayon IM, Miro B, Kalladan R, Reza-Hajirezai M, Oane RH, et al. Variation in primary metabolites in parental and near-isogenic lines of the QTL qDTY12.1: altered roots and flag leaves but similar spikelets of rice under drought. Mol Breed. 2015;35:1–25.

36. Djanaguiraman M, Perumal R, Jagadish SVK, Ciampitti IA, Welti R, Prasad PVV. Sensitivity of sorghum pollen and pistil to high-temperature stress. Plant Cell Environ. 2018;41:1065–82.

37. Do PT, Drechsel O, Heyer AG, Hincha DK, Zuther E. Changes in free polyamine levels, expression of polyamine biosynthesis genes, and performance of rice cultivars under salt stress: a comparison with responses to drought. Front Plant Sci. 2014;5:182.

38. Do PT, Degenkolbe T, Erban A, Heyer AG, Kopka J, Köhl KI, et al. Dissecting rice polyamine metabolism under controlled long-term drought stress. PLoS ONE. 2013;8:e60325.

39. Esposito S. Nitrogen assimilation, abiotic stress and glucose 6-phosphate dehydrogenase: The full circle of reductants. Plants. 2016;5:24.

40. Ioku K, Terao J, Nakatani N. Antioxidative activity of arbutin in a solution and liposomal suspension. Biosci Biotechnol Biochem. 1992;56:1658–9.

41. Hincha DK, Oliver AE, Crowe JH. Lipid composition determines the effects of arbutin on the stability of membranes. Biophys J. 1999;77:2024–34.

42. Sprenger H, Erban A, Seddig S, Rudack K, Thalhammer A, Le MQ, et al. Metabolite and transcript markers for the prediction of potato drought tolerance. Plant Biotechnol J. 2018;16:939–50.

43. Quan N, Anh L, Khang D, Tuyen P, Toan N, Minh T, et al. Involvement of secondary metabolites in response to drought stress of rice (*Oryza sativa* L.). Agriculture. 2016;6:23.

44. Henry A, Gowda VRP, Torres RO, McNally KL, Serraj R. Variation in root system architecture and drought response in rice (*Oryza sativa*): Phenotyping of the OryzaSNP panel in rainfed lowland fields. Field Crop Res. 2011;120:205–14.

45. Kumar A, Dixit S, Ram T, Yadaw RB, Mishra KK, Mandal NP. Breeding high-yielding drought-tolerant rice: Genetic variations and conventional and molecular approaches. J Exp Bot. 2014;65:6265–78.

46. Tenorio FA, Ye C, Redoña E, Sierra S, Laza M, Argayoso MA. Screening rice genetic resources for heat tolerance. SABRAO J Breed Genet. 2013;45:371–81.

47. Cao YY, Chen YH, Chen MX, Wang ZQ, Wu CF, Bian XC, et al. Growth characteristics and endosperm structure of superior and inferior spikelets of indica rice under high-temperature stress. Biol Plant. 2016;60:532–42.

48. Lawas LMF, Bheemanahalli R, Solis CA, Jagadish SVK. Sheathed panicle phenotype (cv. Sathi) maintains normal spikelet fertility and grain filling under prolonged heat stress in rice. Crop Sci. 2018;58:1693–705.

49. Chen T, Xu Y, Wang J, Wang Z, Yang J, Zhang J. Polyamines and ethylene interact in rice grains in response to soil drying during grain filling. J Exp Bot. 2013;64:2523–38.

50. Erban A, Schauer N, Fernie AR, Kopka J. Nonsupervised Construction and Application of Mass Spectral and Retention Time Index Libraries From Time-of-Flight Gas Chromatography-Mass Spectrometry Metabolite Profiles. In: Weckwerth W, editor. Methods Mol Biol. Totowa, NJ: Humana Press Inc.; 2007. p. 19–39.

51. Luedemann A, Strassburg K, Erban A, Kopka J. TagFinder for the quantitative analysis of gas chromatography - Mass spectrometry (GC-MS)-based metabolite profiling experiments. Bioinformatics. 2008;24:732–7.

52. Kopka J, Schauer N, Krueger S, Birkemeyer C, Usadel B, Bergmüller E, et al. GMD@CSB.DB: The Golm metabolome database. Bioinformatics. 2005;21:1635–8.

53. Hummel J, N. S, Bölling C, Schmidt S, Walther W, Kopka J. Mass Spectral Search and Analysis Using the Golm Metabolome Database. In: Wolfram W, Günter K, editors. Handb Plant Metabolomics. Wiley-VCH Verlag GmbH & Co. KGaA; 2013. p. 321–43.

54. EMBL-EBI MetaboLights (DOI: 10.1093/nar/gks1004. PubMed PMID: 23109552) with the identifier MTBLS801

55. R Core Team. R: A language and environment for statistical computing. Vienna, Austria: R Foundattion for Statistical Computing; 2017. Available from: https://www.r-project.org/

56. RStudio Team. RStudio: Integrated Development Environment for R. Boston, MA: RStudio, Inc.; 2016. Available from: http://www.rstudio.com/

57. Stacklies W, Redestig H, Scholz M, Walther D, Selbig J. pcaMethods - A bioconductor package providing PCA methods for incomplete data. Bioinformatics. 2007;23:1164–7.

58. De Livera AM, Olshansky M, Speed TP. Statistical Analysis of Metabolomics Data. In: Roessner U, Dias DA, editors. Metabolomics Tools Nat Prod Discov Methods Protoc. Totowa, NJ: Humana Press; 2013. p. 291–307.

59. Lisec J, Römisch-Margl L, Nikoloski Z, Piepho HP, Giavalisco P, Selbig J, et al. Corn hybrids display lower metabolite variability and complex metabolite inheritance patterns. Plant J. 2011;68:326–36.

60. GitHub (https://github.com/llawas/Rice_HxD_Metabolomics)

Figure 1

Click here to access/download;Figure;Figure 1_HxD_stress.pdf

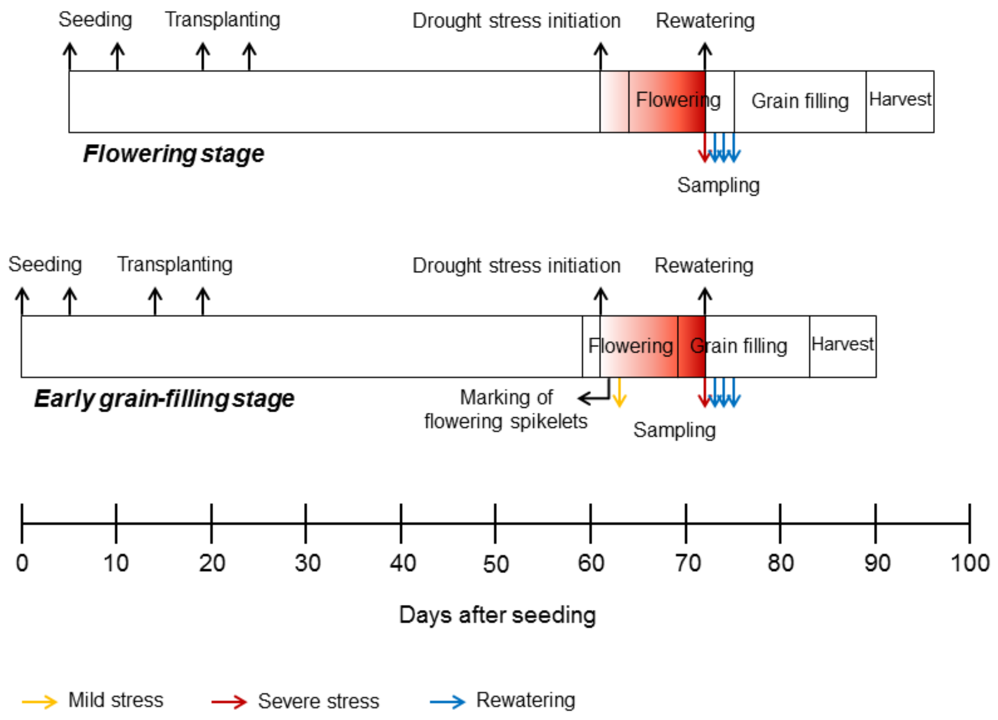

Figure 2

Click here to access/download;Figure;Figure 2_HxD_stress.pdf

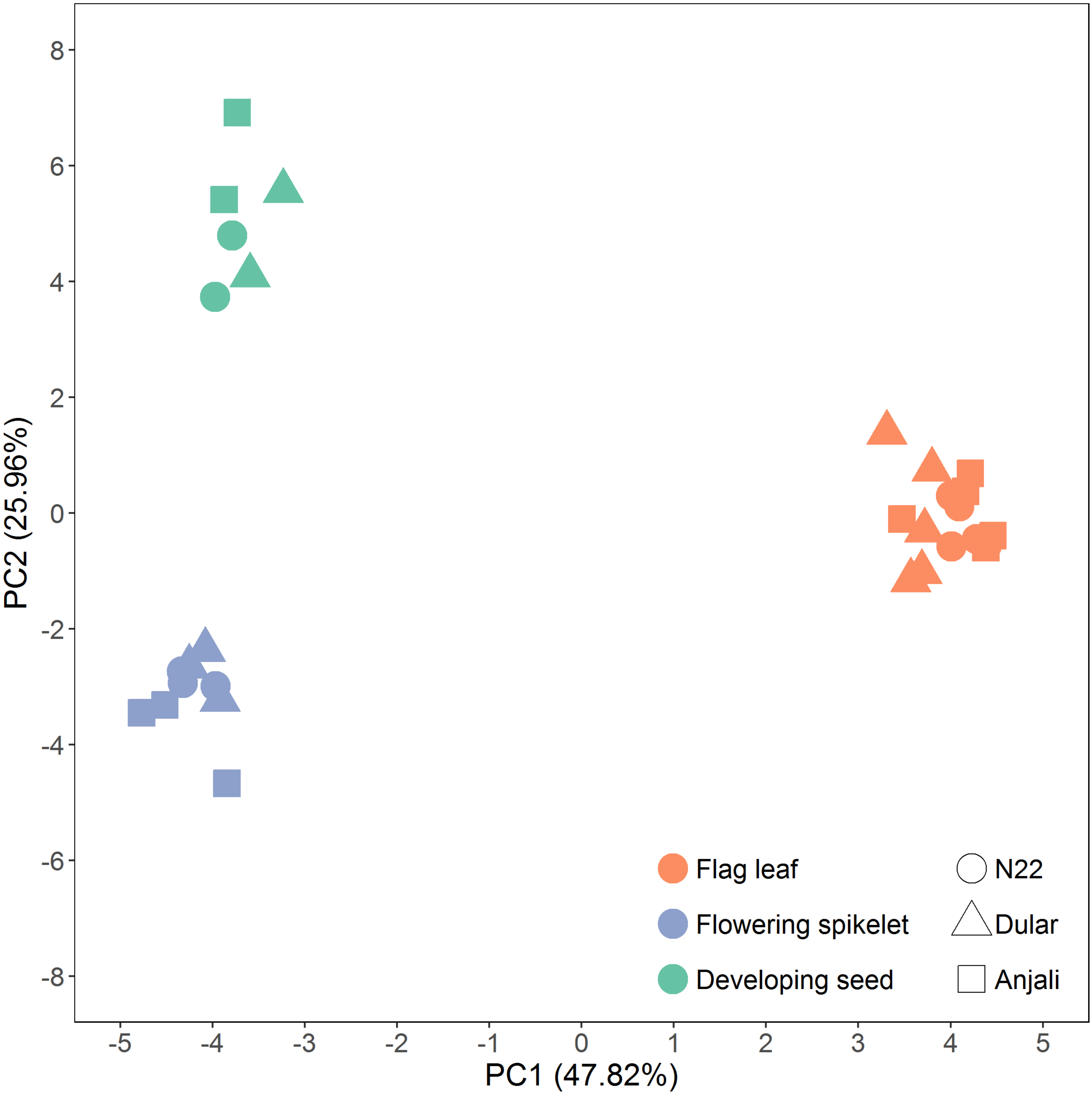

Figure 3

Click here to access/download;Figure;Figure 3_HxD_stress.pdf

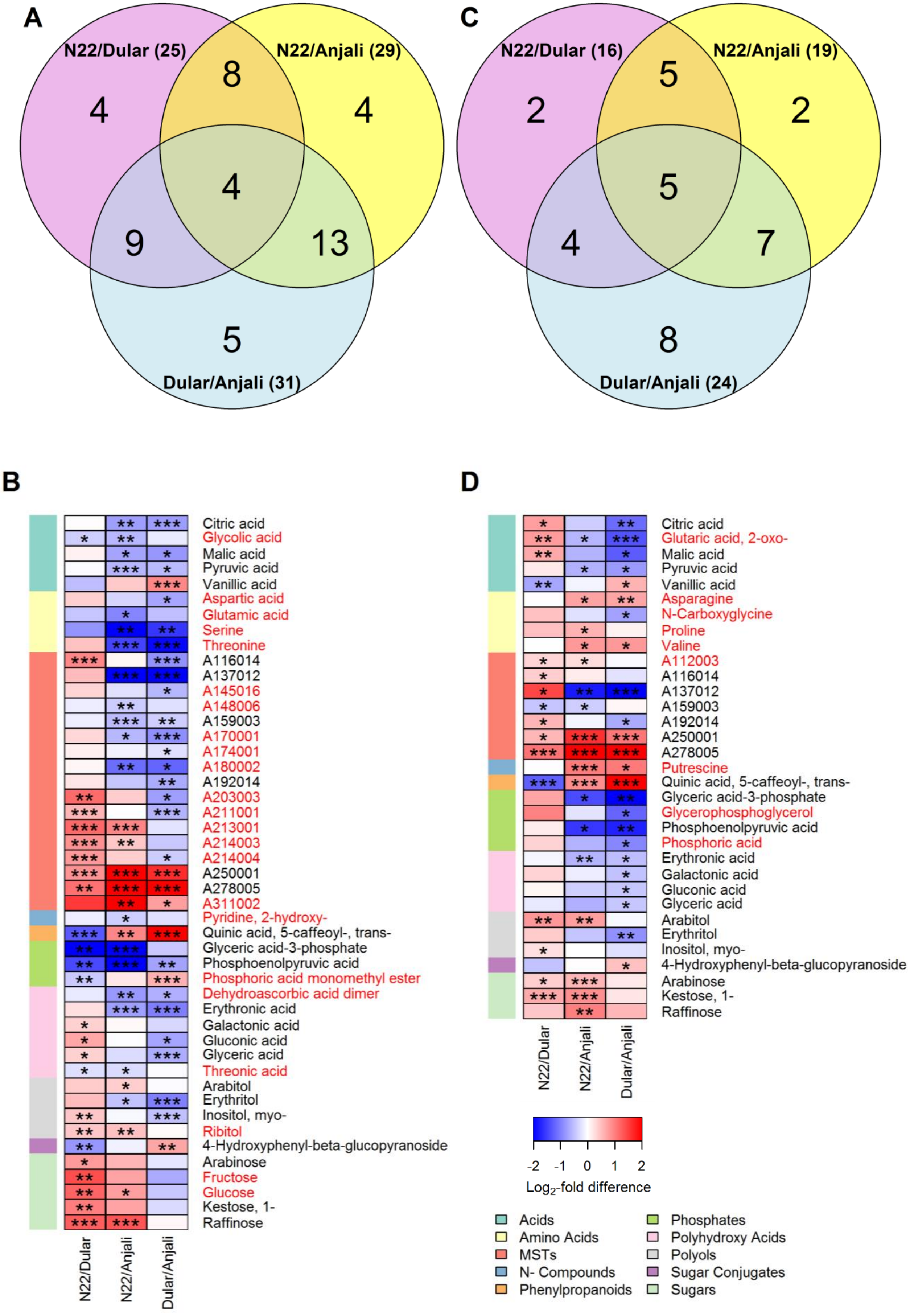

Figure 4

Click here to access/download;Figure;Figure 4_HxD_stress.pdf

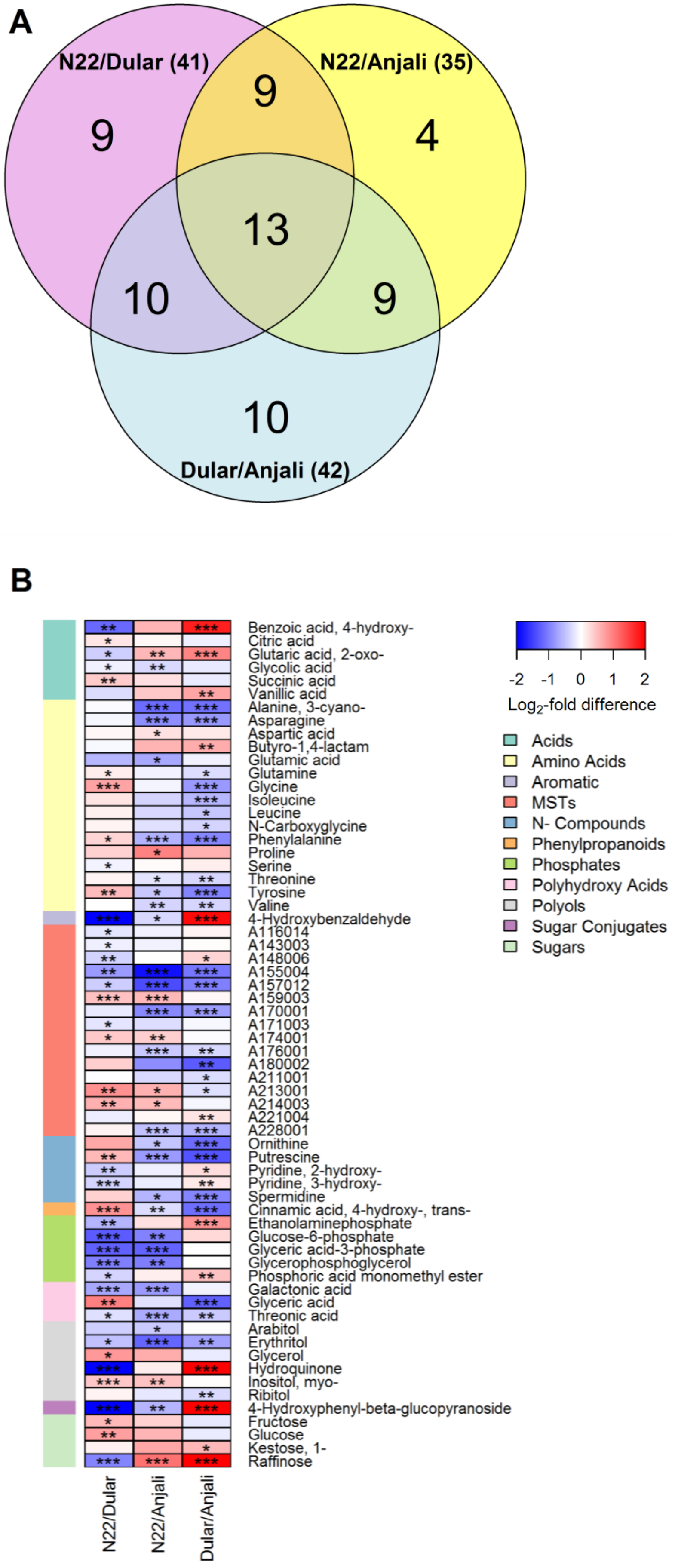

Figure 5

Click here to access/download;Figure;Figure 5_HxD_stress.pdf

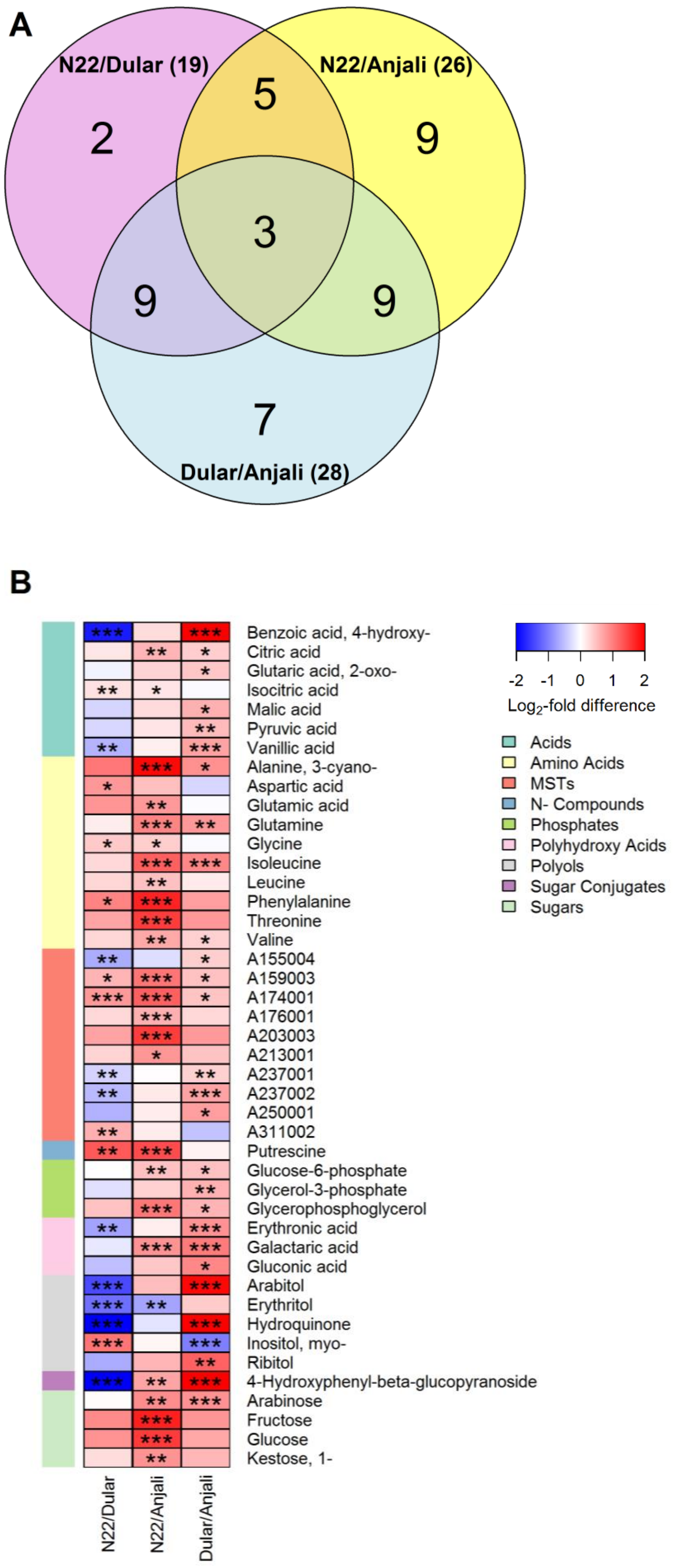

Figure 6

Click here to access/download;Figure;Figure 6_HxD_stress.pdf

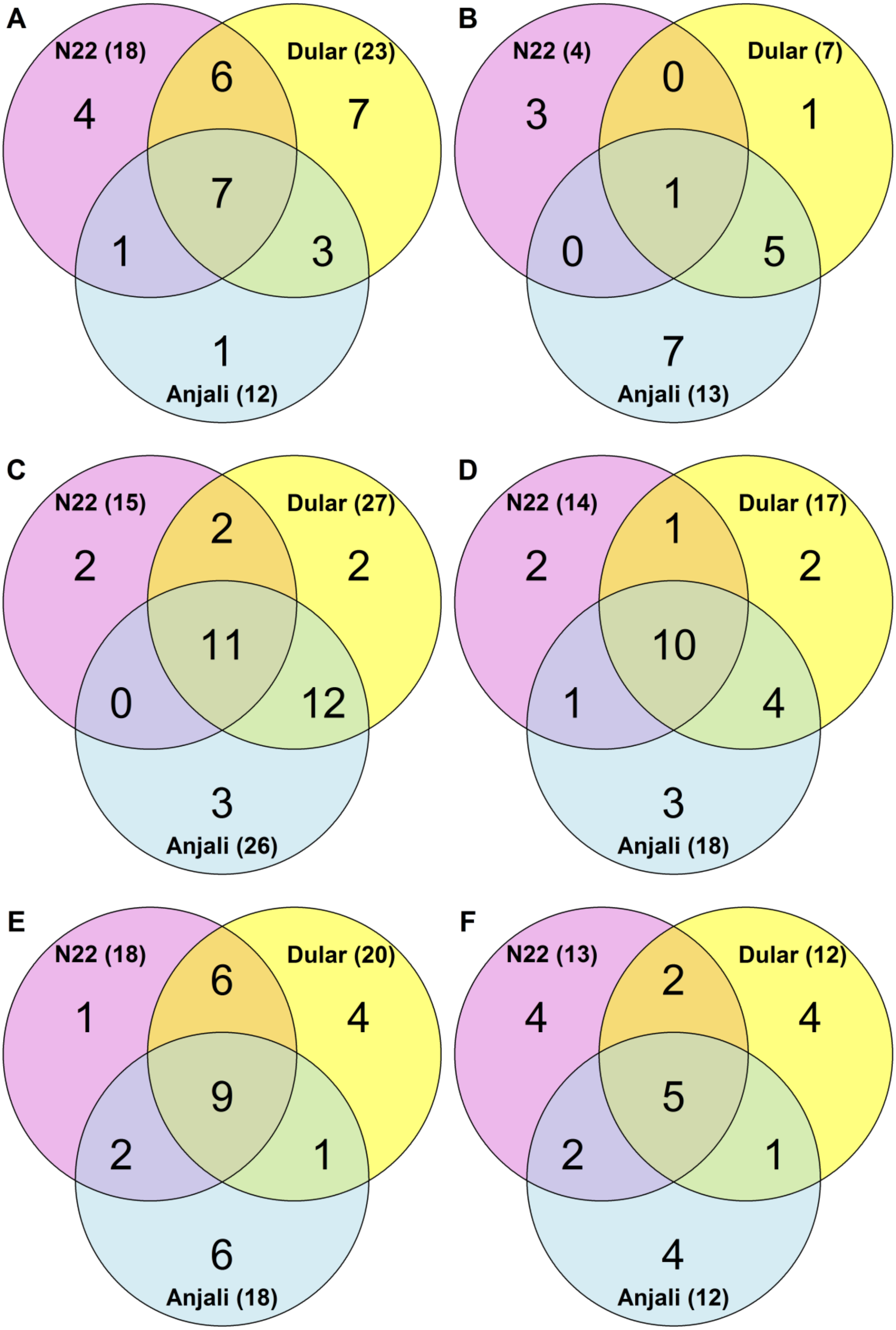

Figure 7

Click here to access/download;Figure;Figure 7_HxD_stress_final_revised.pdf

N22	Dular	Anjali	N22∩Dular	N22∩Anjali	Dular∩Anjali	N22∩Dular∩Anjali
Mild stress, Increase						
Glutamine	Isoleucine	Arabitol	Glutamic acid	A170001	Aspartic acid	Leucine
Serine	Hexadecanoic acid		A137012		Octadecanoic acid	Phenylalanine
A155004	A143003		A211001		A250001	Proline
Chlorogenic acid	A214004		Putrescine			Threonine
	A237001		Raffinose			Valine
	Glycerol		Sucrose			Erythritol
	Arabinose					Inositol, myo-
Mild stress, Decrease						
Succinic acid	A148006	Glutaric acid, 2-oxo-			Citric acid	Malic acid
Sinapic acid, trans-		Pyruvic acid			Glycolic acid	
Glucose-6-phosphate		A116014			Isocitric acid	
		A159003			Glycerate-3-phosphate	
		A180002			Phosphoenolpyruvate	
		A192014				
		Pyridine, 2-hydroxy-				
Severe stress, Increase						
Glutamic acid	*Aspartic acid*	*A170001*	*Valine*		*Glycine*	*N-Carboxyglycine*
Arbutin	Erythronic acid	A213001	*A137012*		*Isoleucine*	**Phenylalanine**
		Ribitol			Leucine	**Proline**
					Serine	**Threonine**
					A203003	**A155004**
					A237001	*A211001*
					A311002	*A250001*
					Glycerol	*Putrescine*
					Inositol, myo-	*Arabitol*
					Arabinose	*Erythritol*
					Fructose	**Raffinose**
					Glucose	
Severe stress, Decrease						
Phosphoric acid	*A148006*	A116014	Glutaric acid, 2-oxo-	Glyceric acid	*Glycolic acid*	**Citric acid**
Galactonic acid	A171003	Chlorogenic acid			*Glycerate-3-phosphate*	**Isocitric acid**
		Galactaric acid			*Phosphoenolpyruvate*	*Malic acid*
					Dehydroascorbic acid dimer	*Pyruvic acid*
						**Succinic acid**
						**A192014**
						Pyridine, 2-hydroxy-
						Pyridine, 3-hydroxy-
						Glucose-6-phosphate
						Monomethyl phosphate

Figure 8
Click here to access/download;Figure;Figure 8_HxD_stress_revised.pdf

N22	Dular	Anjali	N22∩Dular	N22∩Anjali	Dular∩Anjali	N22∩Dular∩Anjali
			Increase			
Vanillic acid	*Aspartic acid*	*Serine*	*Glycine*	*Inositol, myo-*	*Arabitol*	*Isoleucine*
	Glutamic acid	*A211001*	*N-Carboxyglycine*	*Arabinose*		**Phenylalanine**
	A311002	*A250001*	*A137012*			**Proline**
	Fructose	*Putrescine*	*A237001*			**Threonine**
		Erythritol	Ribitol			*Valine*
		Glycerol	*Glucose*			**A155004**
						A170001
						A203003
						**Raffinose**
			Decrease			
Malic acid	A112003	Glutaric acid, 2-oxo-	*Pyruvic acid*	*Pyridine, 2-hydroxy-*	*Glycolic acid*	**Citric acid**
A278005	A145016	*Glycerate-3-phosphate*	*A148006*	*Glucose-6-phosphate*		**Isocitric acid**
Phosphoric acid	A147011	*Phosphoenolpyruvate*				**Succinic acid**
Ascorbic acid	Sucrose	Galactonic acid				**A192014**
						Dehydroascorbic acid dimer

Figure 9

Click here to access/download;Figure;Figure 9_HxD_stress.pdf

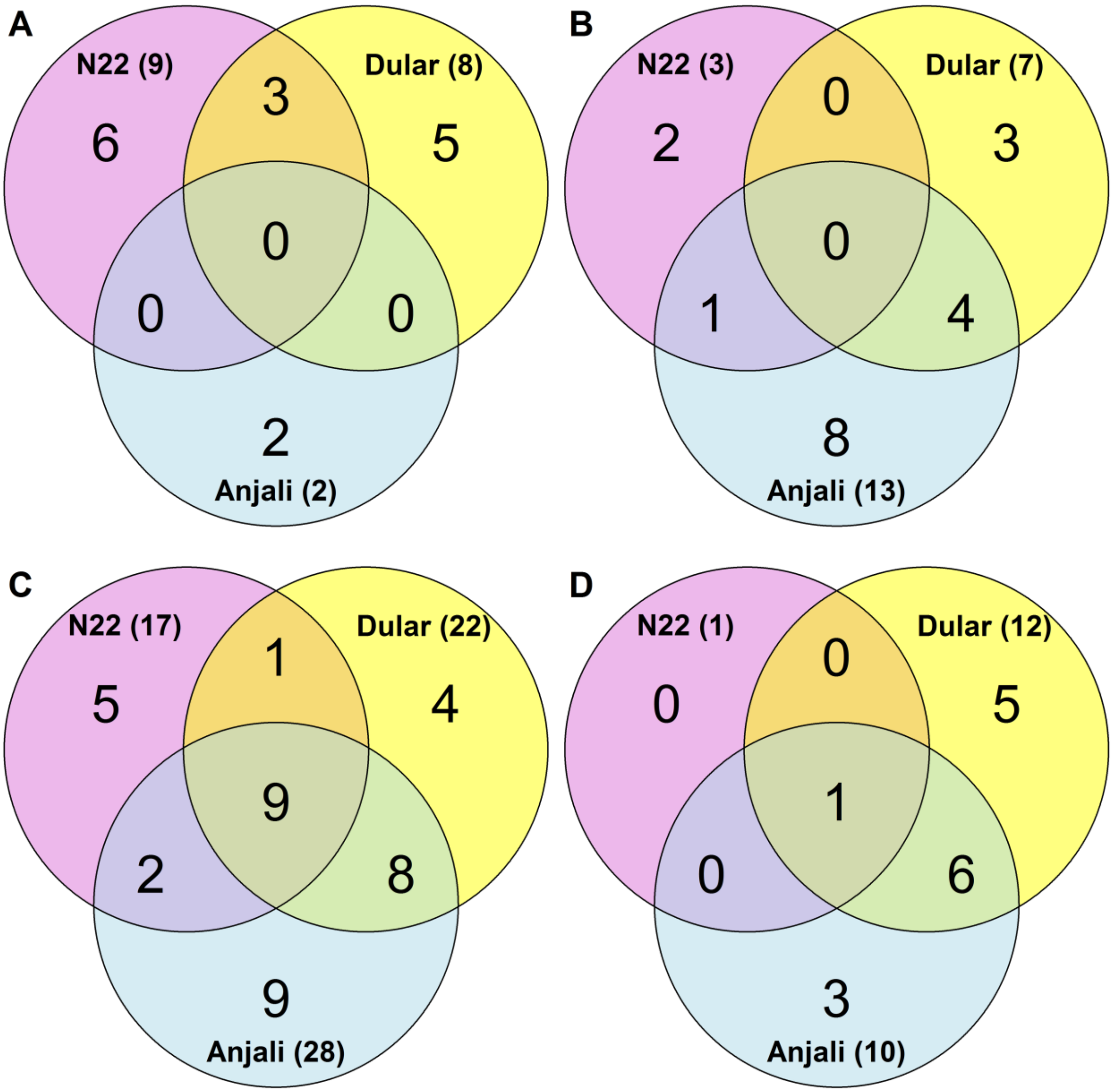

Figure 10

Click here to access/download;Figure;Figure 10_HxD_stress_revised.pdf

N22	Dular	Anjali	N22∩Dular	N22∩Anjali	Dular∩Anjali	N22∩Dular∩Anjali
			Mild stress, Increase			
Glycolic acid	Malic acid	Isoleucine	A155004			
Alanine, 3-cyano-	A203003	Proline	A170001			
A211001	A214003		A237002			
Spermidine	Erythritol					
Glycerophosphoglycerol	Kestose, 1-					
Raffinose						
			Mild stress, Decrease			
Threonine	Asparagine	N-Carboxyglycine		Threonic acid	A157012	
Phosphoric acid	Glutamine	Phenylalanine			A171003	
	Arbutin	Serine			A174001	
		A148006			Pyridine, 2-hydroxy-	
		Ornithine				
		Pyridine, 3-hydroxy-				
		Cinnamic acid, 4-hydroxy-, trans-				
		Arabinose				
			Severe stress, Increase			
Serine	Glycine	Pyruvic acid	Malic acid	Spermidine	*Phenylalanine*	**Isoleucine**
A116014	Valine	Butyro-1,4-lactam		Raffinose	Tyrosine	Leucine
A211001	Ribitol	Proline			A203003	*Threonine*
Glycerophosphoglycerol	Arabinose	A214003			A213001	*A155004*
Monomethyl phosphate		A250001			A237001	*A170001*
		A311002			Glycerol-3-phosphate	*A237002*
		Putrescine			Fructose	*Arabitol*
		Gluconic acid			Glucose	Erythritol
		Kestose, 1-				Inositol, myo-
			Severe stress, Decrease			
	Benzoic acid, 4-hydroxy-	N-Carboxyglycine			A145008	A148006
	4-Hydroxy-benzaldehyde	A176001			A159003	
	Pyridine, 3-hydroxy-	Phosphoric acid			A171003	
	Glucose-6-phosphate				A174001	
	Hydroquinone				Pyridine, 2-hydroxy-	
					Arbutin	

Figure 11

Click here to access/download;Figure;Figure 11_HxD_stress_revised.pdf

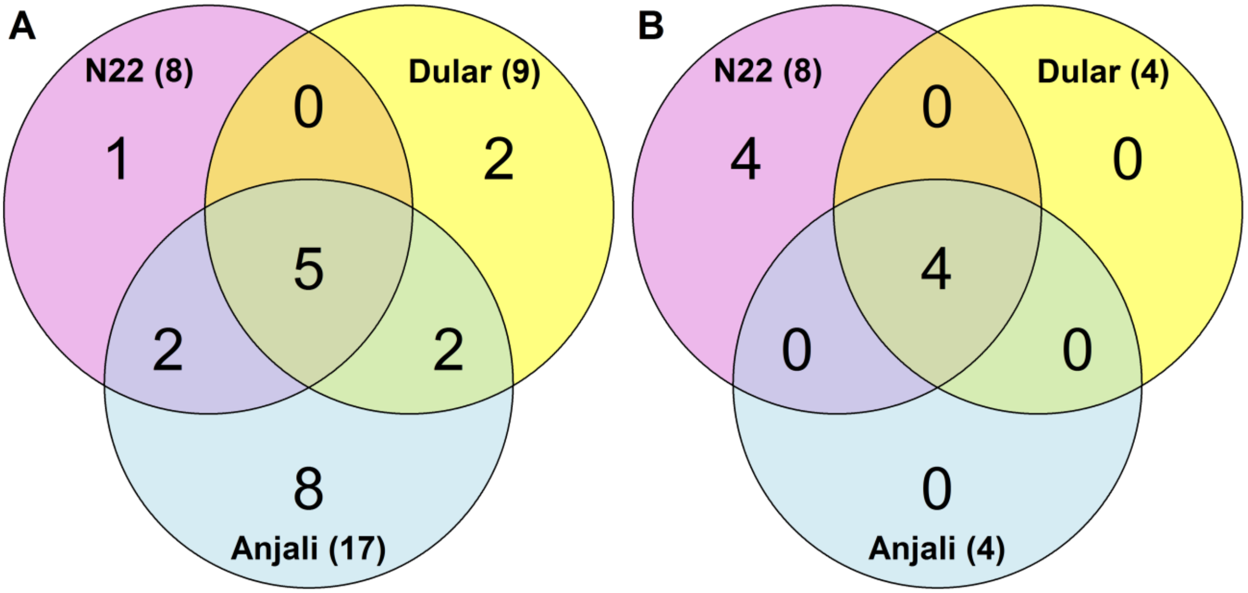

C

N22	Dular	Anjali	N22∩Dular	N22∩Anjali	Dular∩Anjali	N22∩Dular∩Anjali
			Increase			
Arabitol	Aspartic acid	Glutamine		Glycine	A228001	Alanine, 3-cyano-
	Raffinose	N-Carboxyglycine		Arbutin	Galactaric acid	**Isoleucine**
		Threonine				*Phenylalanine*
		A155004				A159003
		A170001				A174001
		A176001				
		Pyridine, 2-hydroxy-				
		Arabinose				
			Decrease			
A145016						Succinic acid
A147011						*Glucose-6-phosphate*
A213001						Erythronic acid
Threonic acid						Erythritol

Figure 12

Click here to access/download;Figure;Figure 12_HxD_stress.pdf

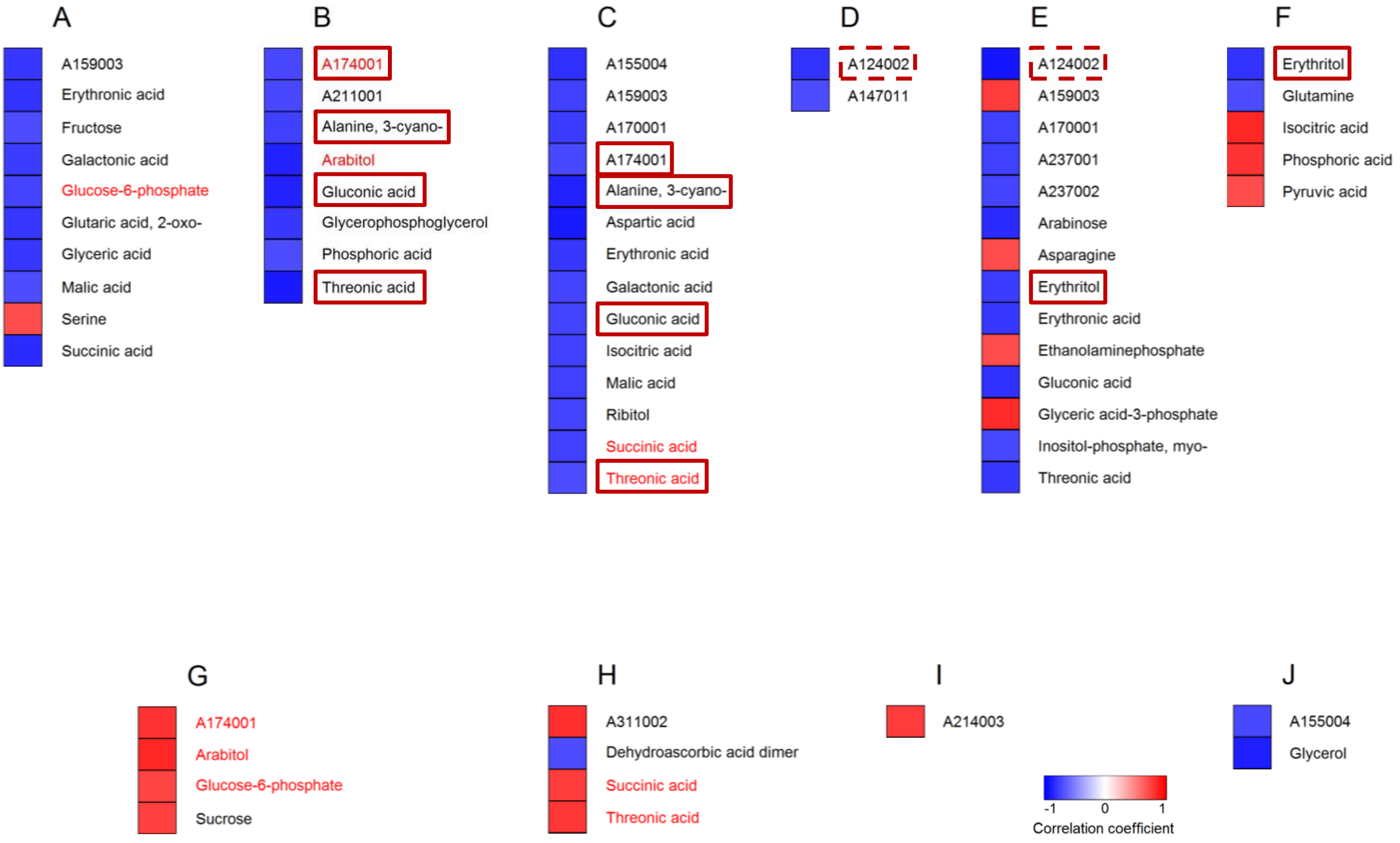

Figure 13

Click here to access/download;Figure;Figure 13_HxD_stress.pdf

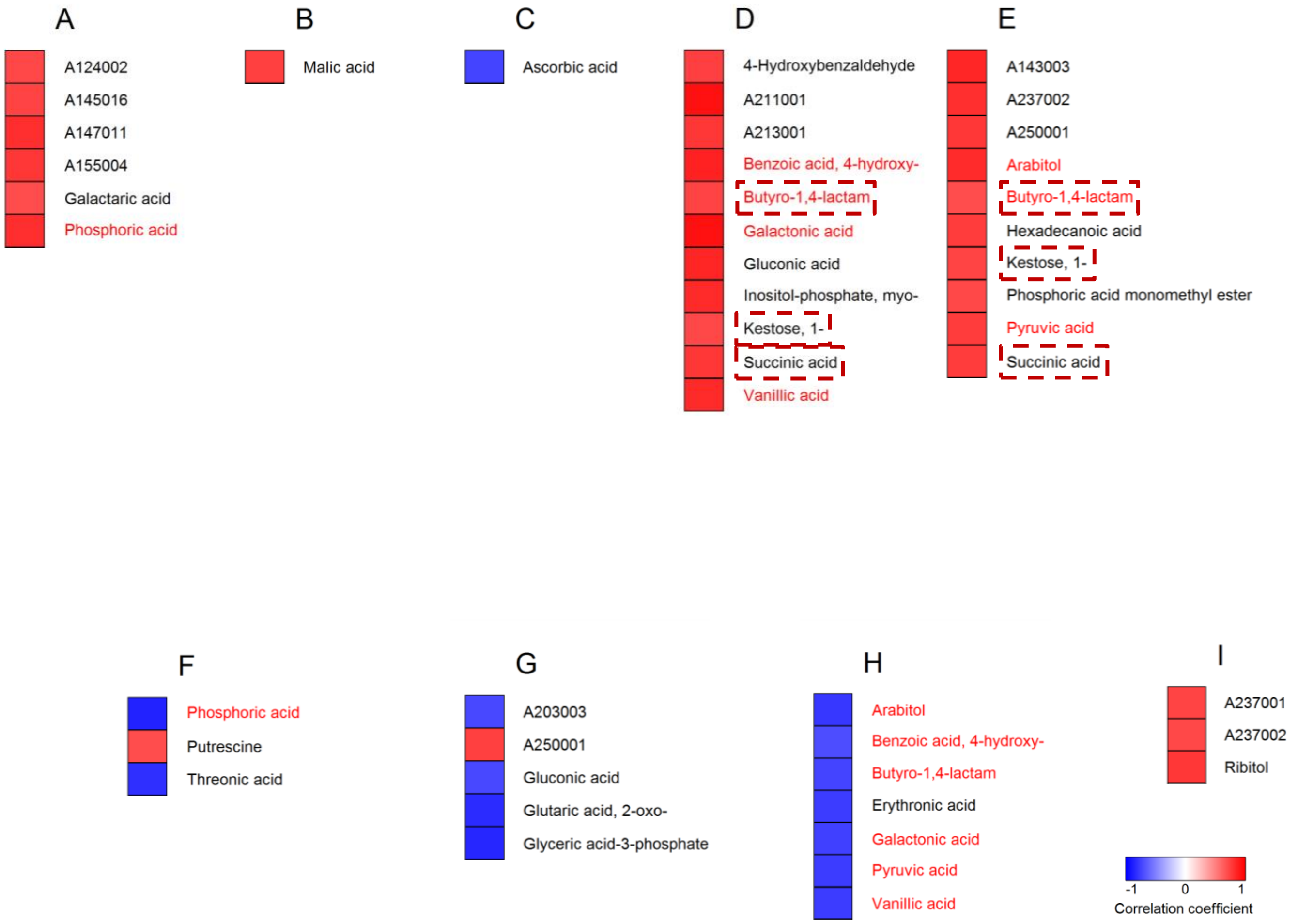

Additional file 1

Click here to access/download
Supplementary Material
Additional file 1_HxD_stress.xlsx

Additional file 2

Click here to access/download
Supplementary Material
Additional file 2_HxD_stress_wLeg.pdf

Additional file 3

Click here to access/download
Supplementary Material
Additional file 3_HxD_stress_wLeg.pdf
