# Supplementary material for: Metabolic responses of rice cultivars with different tolerance to combined drought and heat stress under field conditions
Source: Gigascience. 2019 May 13;8(5):giz050. doi: 10.1093/gigascience/giz050 (PMC6511916; doi:10.1093/gigascience/giz050)
Supplement: Supplemental Files [file giz050_supplemental_files.zip › Additional file 3_HxD_stress_wLeg.pdf]

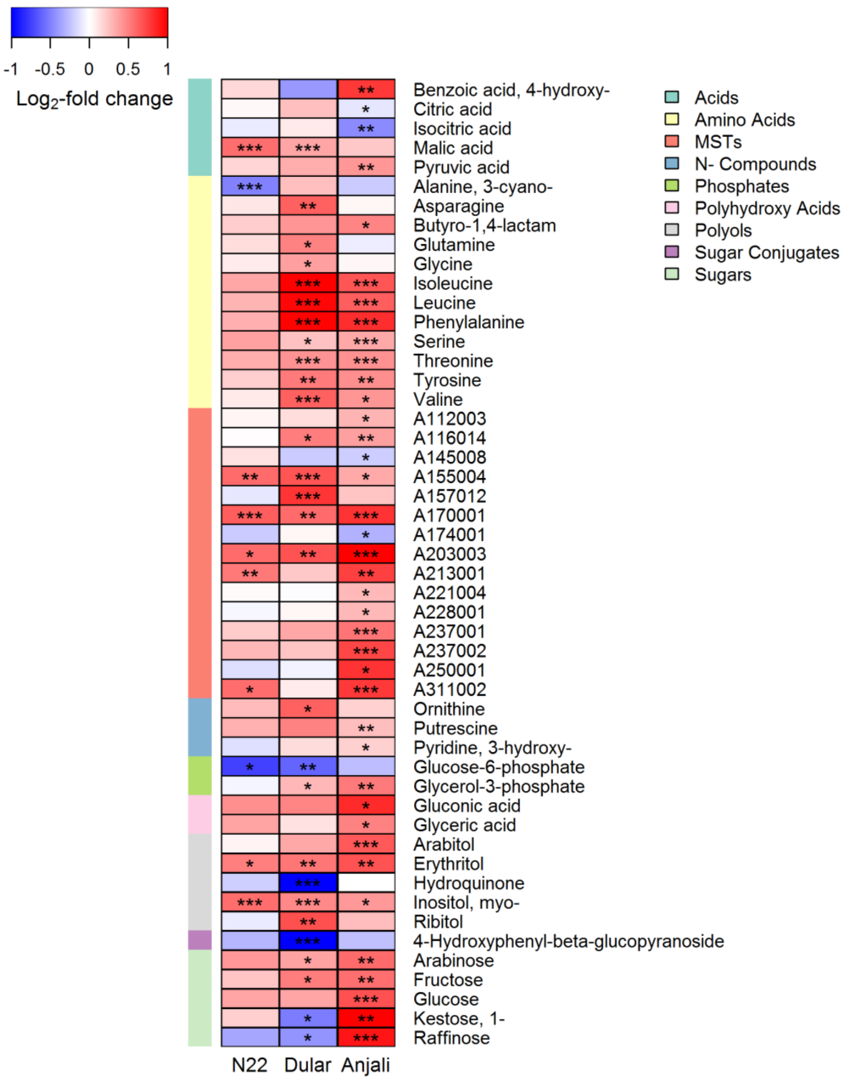


**Additional file 3 (PDF). Metabolites with different abundance during mild and severe drought and heat stress in flowering spikelets**

The heat map displays all metabolites in flowering spikelets that showed a significant (Mann-Whitney-Wilcoxon test, $P < 0.05$) difference in abundance between mild and severe combined drought and heat stress during the flowering stage. The level of significance is indicated for each metabolite and cultivar by asterisks (* $P < 0.05$; ** $P < 0.01$; *** $P < 0.001$) and the $\log_2$-fold difference is indicated by the color code.
