# Supplementary material for: Metabolic responses of rice cultivars with different tolerance to combined drought and heat stress under field conditions
Source: Gigascience. 2019 May 13;8(5):giz050. doi: 10.1093/gigascience/giz050 (PMC6511916; doi:10.1093/gigascience/giz050)
Supplement: Response_to_Reviewer_Comments_Original_Submission.pdf [file giz050_response_to_reviewer_comments_original_submission.pdf]

Author's Response To Reviewer Comments

Close

Reviewer reports:
Reviewer #1: In this manuscript the authors performed metabolic profiling analysis of three rice cultivars grown in field conditions. Drought was applied during early flowering or grain filling. The authors studied the metabolic differences induced by drought and the different responses of the three cultivars. This is a follow up study from the same authors where other agronomic measurements were taken. In this paper the authors make correlations analysis between metabolite levels and yield reduction under stress.
I wholeheartedly agree with the authors on the necessity of conducting this type of analysis in plants grown in field conditions and particularly trying to induce several stresses.
My main concern about the paper is about the way the idea of combined drought/heat stress is presented. How plants respond to these two stresses is a very interesting subject and a lot of research is currently going on about how climate change will impact certain areas with a combination or both stresses while in other areas only high temperature will be the predominant factor. In the paper the authors acknowledge the fact that their experimental design only allows them to control drought stress by means of controlling irrigation. Temperature is not controlled in their experimental setup. However, it should be more clear how why the authors consider that the temperature at which they grew the plants in the different seasons is considered stressful for the cultivars used in the experiment. It would be important to clarify this since the main idea of the paper is to study metabolic differences of cultivars grown under a combination of heat and drought stress.

Response: We apologize if we have not made this point sufficiently clear. We have now added information under Data Description to describe how we showed in our earlier publication that the plants were indeed heat stressed. The modified text now reads as follows:
We performed three field experiments in 2013, 2014 and 2015 at the International Rice Research Institute (IRRI) in the Philippines during the dry season (late April to early May, the hottest time of the year) including the three rice cultivars N22, Dular and Anjali that differ in their response to drought and heat stress. Plants were either grown in flooded paddies throughout their life cycle, or were drought stressed either during flowering or early grain filling. At the end of the stress period, plants were rewatered to allow seed set. Even under the prevailing climatic conditions, drought induced an increase in both panicle and canopy-air temperature due to the lack of transpirational cooling [17]. Soil and plant water status, air and plant temperature, leaf and panicle transpiration rates, along with seed yield and seed quality were monitored throughout the experiments. The results showed that canopy-air temperature frequently rose above 33°C under drought stress, the temperature considered the threshold for the induction of spikelet sterility of rice in the field. This was accompanied by significant reductions in grain yield [17].

I have no concerns about the data analysis presented by the authors, only some suggestions about the best way to present it:

- Please keep font size and type uniform across figures.
Response: We thank the reviewer for pointing this out. We have revised Figures 7, 8, 10 and 11 accordingly.

- With so many venn diagrams and tables it might be useful to consider something like https://github.com/hms-dbmi/UpSetR or any other approach that can help visualize intersections between the different genotypes and treatments more easily.
Response: We are aware of these approaches but decided against using them for this paper. These visualization tools are very useful e.g. for transcriptomic data, where hundreds of genes have to be classified. However, the number of metabolites detectable by GC-MS is still comparatively small so that relevant metabolites in the different intersections could just be listed individually. We find this more appropriate because in this way the metabolite identities are immediately obvious to the reader, while with an approach such as UpSetR the reader would need to refer to an additional supplemental table to see the identity of the metabolites.

- Related to the point above, it would be useful to combine tables and ven diagrams that referr to the same dataset. E.g. Figure 9 and 10.
Response: We have combined Venn diagrams and heatmaps/tables as far as possible. However, in the case of Figures 6 and 7, and 9 and 10 the combination would require to reduce the size of the single panels to a degree that they would no longer be readable. We would therefore strongly prefer to leave these figures unaltered.

Reviewer #2: The authors conducted metabolome analysis of 3 rice cultivars with different drought tolerance. The samples are flag leaves, flowering spikelets, and developing grains collected from rice plants grown in the paddy field under combined drought and heat stress conditions. The sampling was conducted over 3 consecutive years. Based on the datasets acquired by GC-MS from these samples, the authors analyzed the metabolic responses of respective organs, and found several metabolic markers linked to the stress tolerance, yield and grain quality. Although it remains still unknown whether these marker metabolites provide drought tolerance to plants, these markers, especially deduce from the analysis of samples from plants grown under well-irrigated control conditions, might be useful for breeding of rice. Although in this experiment quite different cultivars are used (aus, indica), further analysis using more cultivars selected by considering their genetic relationship
might reveal more suited markers for breeding.

Close
