# Supplementary material for: Metabolic responses of rice cultivars with different tolerance to combined drought and heat stress under field conditions
Source: Gigascience. 2019 May 13;8(5):giz050. doi: 10.1093/gigascience/giz050 (PMC6511916; doi:10.1093/gigascience/giz050)
Supplement: Response_to_Reviewer_Comments_Revision_1.pdf [file giz050_response_to_reviewer_comments_revision_1.pdf]

Author's Response To Reviewer Comments

Close

Dear Nicole,

I have made all requested editorial changes. I hope I did not miss anything.

Thanks for your help and best regards

Dirk

Close
