# Supplementary material for: Metabolic responses of rice cultivars with different tolerance to combined drought and heat stress under field conditions
Source: Gigascience. 2019 May 13;8(5):giz050. doi: 10.1093/gigascience/giz050 (PMC6511916; doi:10.1093/gigascience/giz050)
Supplement: Reviewer_1_Report_Original_Submission -- Ruben Rellan Alvarez — 2/14/2019 Reviewed [file giz050_reviewer_1_report_original_submission.pdf]

Reviewer Report

Title: Metabolic responses of rice cultivars with different tolerance to combined drought and heat stress under field conditions

Version: Original Submission **Date:** 2/14/2019

Reviewer name: Ruben Rellan Alvarez

Reviewer Comments to Author:

In this manuscript the authors performed metabolic profiling analysis of three rice cultivars grown in field conditions. Drought was applied during early flowering or grain filling. The authors studied the metabolic differences induced by drought and the different responses of the three cultivars. This is a follow up study from the same authors where other agronomic measurements were taken. In this paper the authors make correlations analysis between metabolite levels and yield reduction under stress.
I wholeheartedly agree with the authors on the necessity of conducting this type of analysis in plants grown in field conditions and particularly trying to induce several stresses.
My main concern about the paper is about the way the idea of combined drought/heat stress is presented. How plants respond to these two stresses is a very interesting subject and a lot of research is currently going on about how climate change will impact certain areas with a combination or both stresses while in other areas only high temperature will be the predominant factor. In the paper the authors acknowledge the fact that their experimental design only allows them to control drought stress by means of controlling irrigation. Temperature is not controlled in their experimental setup. However, it should be more clear how why the authors consider that the temperature at which they grew the plants in the different seasons is considered stressful for the cultivars used in the experiment. It would be important to clarify this since the main idea of the paper is to study metabolic differences of cultivars grown under a combination of heat and drought stress.
I have no concerns about the data analysis presented by the authors, only some suggestions about the best way to present it:
- Please keep font size and type uniform across figures.
- With so many venn diagrams and tables it might be useful to consider something like https://github.com/hms-dbmi/UpSetR or any other approach that can help visualize intersections between the different genotypes and treatments more easily.
- Related to the point above, it would be useful to combine tables and ven diagrams that referr to the same dataset. E.g. Figure 9 and 10.

Methods

Are the methods appropriate to the aims of the study, are they well described, and are necessary controls included? Choose an item.

Conclusions

Are the conclusions adequately supported by the data shown? Choose an item.

Reporting Standards

Does the manuscript adhere to the journal's guidelines on minimum standards of reporting? Choose an item.

Choose an item.

Statistics

Are you able to assess all statistics in the manuscript, including the appropriateness of statistical tests used? Choose an item.

Quality of Written English

Please indicate the quality of language in the manuscript: Choose an item.

Declaration of Competing Interests

Please complete a declaration of competing interests, considering the following questions:

- Have you in the past five years received reimbursements, fees, funding, or salary from an organisation that may in any way gain or lose financially from the publication of this manuscript, either now or in the future?
- Do you hold any stocks or shares in an organisation that may in any way gain or lose financially from the publication of this manuscript, either now or in the future?
- Do you hold or are you currently applying for any patents relating to the content of the manuscript?
- Have you received reimbursements, fees, funding, or salary from an organization that holds or has applied for patents relating to the content of the manuscript?
- Do you have any other financial competing interests?
- Do you have any non-financial competing interests in relation to this paper?

If you can answer no to all of the above, write 'I declare that I have no competing interests' below. If your reply is yes to any, please give details below.

I declare that I have no competing interests'

I agree to the open peer review policy of the journal. I understand that my name will be included on my report to the authors and, if the manuscript is accepted for publication, my named report including any attachments I upload will be posted on the website along with the authors' responses. I agree for my report to be made available under an Open Access Creative Commons CC-BY license (http://creativecommons.org/licenses/by/4.0/). I understand that any comments which I do not wish to be included in my named report can be included as confidential comments to the editors, which will not be published.

I agree to the open peer review policy of the journal

To further support our reviewers, we have joined with Publons, where you can gain additional credit to further highlight your hard work (see: https://publons.com/journal/530/gigascience). On publication of this paper, your review will be automatically added to Publons, you can then choose whether or not to claim your Publons credit. I understand this statement.

Yes Choose an item.
